# Paeoniflorin: a review of its pharmacology, pharmacokinetics and toxicity in diabetes

**DOI:** 10.3389/fphar.2025.1551368

**Published:** 2025-04-07

**Authors:** Xue Ou, Zhijie Yu, Chuanyu Pan, Xi Zheng, Dandan Li, Zhenzhen Qiao, Xiaoyuan Zheng

**Affiliations:** Pharmacy Department, Chongqing Emergency Medical Center, Chongqing University Central Hospital, School of Medicine, Chongqing University, Chongqing, China

**Keywords:** paeoniflorin, diabetes, pathogenesis, pharmacology, pharmacokinetics, toxicity

## Abstract

The escalating global prevalence of diabetes underscores the urgency of addressing its treatment and associated complications. Paeoniflorin, a monoterpenoid glycoside compound, has garnered substantial attention in recent years owing to its potential therapeutic efficacy in diabetes management. Thus, this study aims to systematically overview the pharmacological effects, pharmacokinetics and toxicity of paeoniflorin in diabetes. Plenty of evidences have verified that paeoniflorin improves diabetes and its complication through reducing blood sugar, enhancing insulin sensitivity, regulating gut microbiota and autophagy, restoration of mitochondrial function, regulation of lipid metabolism, anti-inflammation, anti-oxidative stress, inhibition of apoptosis, immune regulation and so on. Paeoniflorin possess the characteristics of rapid absorption, wide distribution, rapid metabolism and renal excretion. Meanwhile, toxicity studies have suggested that paeoniflorin has low acute toxicity, minimal subacute and chronic toxicity, and no genotoxic or mutational toxic effects. In conclusion, this paper systematically elucidates the potential therapeutic application and safety profile of paeoniflorin in diabetes management.

## 1 Introduction

Diabetes is an escalating chronic metabolic disorder globally, primarily characterized by elevated blood glucose levels resulting from inadequate insulin secretion or impaired insulin function (Tokarz et al., 2018; Sims et al., 2021). Persistent hyperglycemia not only precipitates various microvascular complications, such as diabetic nephropathy (DN) and retinopathy, but also elevates the risk of macrovascular complications, including cardiovascular diseases and neuropathy, significantly compromising patients’ quality of life and lifespan (Malik, 2020; Jansson Sigfrids et al., 2021; Hermans et al., 2022; Li X. et al., 2023). Thus, currently diabetes is frequently accompanied by the diabetic nephropathy, diabetic peripheral neuropathy, diabetic wound, diabetic liver injury, diabetic myocardial ischemic injury, diabetic retinopathy and so on. In recent decades, considerable advancements have been achieved in diabetes and its complications’ treatment. However, oral antidiabetic medications and insulin therapy despite effectively control blood glucose, but frequently bring about multiple side effects. Meanwhile, unstable therapeutic outcomes and necessitate long-term usage constrain clinical application seriously (Levy et al., 2019; McAlister et al., 2021; Gorgogietas et al., 2023).

Recently, natural products have increasingly been spotlighted as potential novel strategies for diabetes and its complications’ treatment (Yang X. et al., 2022; Wang et al., 2023a). Notably, Paeoniflorin (PF), a principal bioactive compound in traditional Chinese herbs (Paeonia lactiflora Pall.), has garnered significant attention due to its remarkable hypoglycemic properties, protective effects on pancreatic β-cells, and amelioration of diabetic complications (Luo W. et al., 2023; Liang et al., 2024; Wu et al., 2024). Study indicated that hypoglycemic effects of PF *via* enhancing insulin sensitivity, stimulating insulin secretion, inhibiting hepatic glycogenolysis, and augmenting muscle glucose uptake (Gu et al., 2016; Yao et al., 2021; Zhang et al., 2023a; Zhang et al., 2023b). Furthermore, PF could improve pancreatic β-cells *via* antioxidative, anti-inflammatory, and antifibrotic pathways, mitigating the incidence and progression of diabetic complications (Zhang et al., 2020; Liu et al., 2023a). As research into the mechanism of action of PF intensifies, an increasing number of clinical and experimental studies have validated its potential therapeutic value in managing diabetes and its complications. Multiple studies have demonstrated that PF can markedly lower blood glucose levels, enhance pancreatic function, and attenuate the onset and progression of diabetic nephropathy, while also displaying favorable safety and tolerability profiles (Bayan et al., 2022).

**FIGURE 1:**
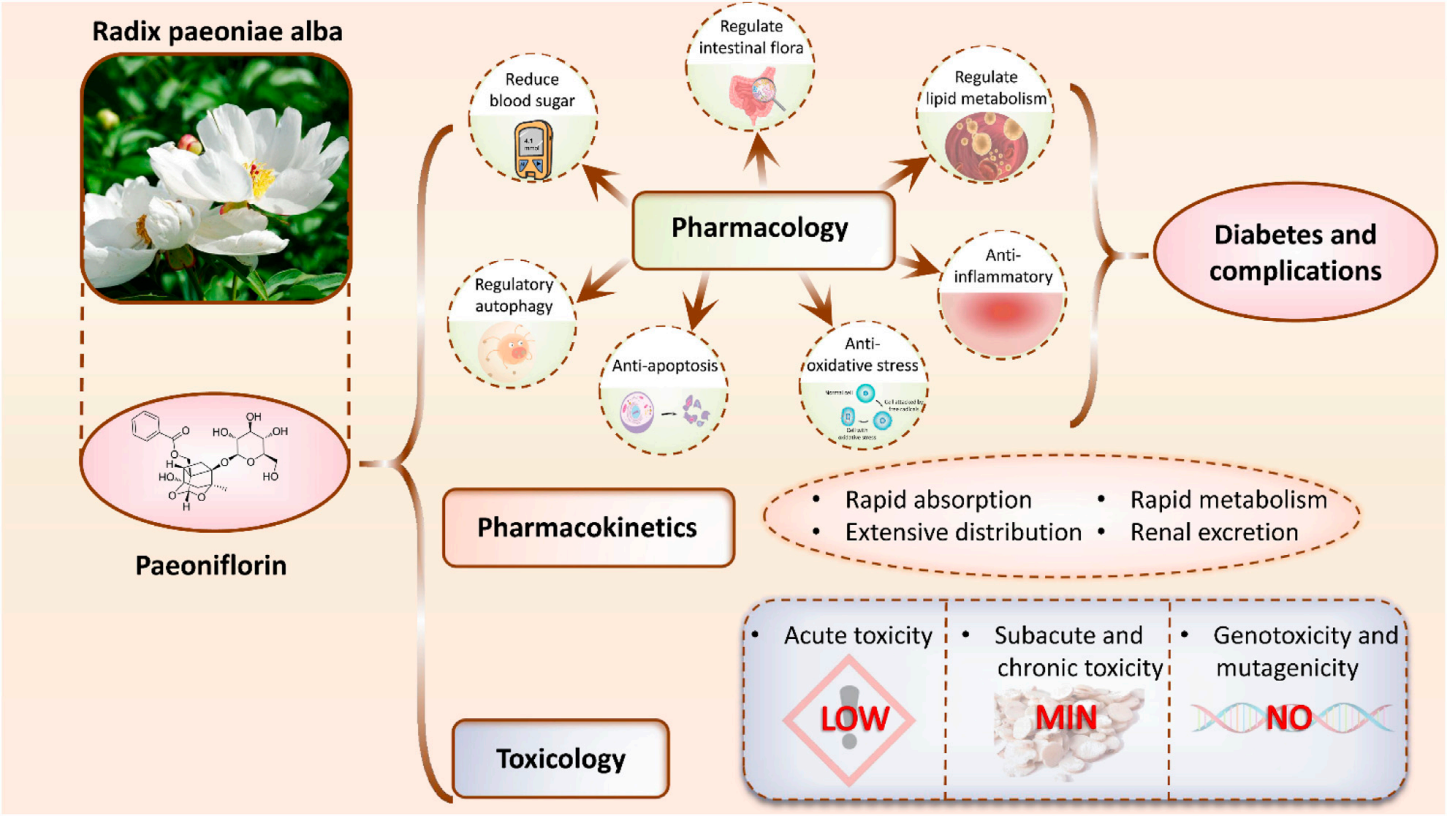
Mechanisms of PF in the treatment of diabetes and its complications. PF exerts therapeutic effects by lowering blood glucose levels, modulating gut microbiota, regulating lipid metabolism, and exhibiting anti-inflammatory, antioxidant, anti-apoptotic, and autophagy-regulating properties. In addition, its pharmacokinetics and toxicological profiles are summarized, highlighting the compound’s potential as a therapeutic agent for diabetes and its associated complications.

Nonetheless, numerous questions persist concerning PF’s mechanism of action in diabetes and its complications’ treatment, including its specific targets, action pathways, and potential interactions with other antidiabetic medications, necessitating further exploration. Therefore, this article aims to comprehensively summarize and analyze the research progress on PF’s role in treating diabetes and its complications, furnishing a scientific foundation for its expanded clinical application and introducing novel insights and approaches to refine and innovate diabetes and its complications’ therapeutic strategies (Figure 1). All abbreviations along with their full forms as shown in (Table 1).

**TABLE 1:** List of abbreviations and the corresponding full terms.

Abbreviation	Full terms
3-NT	3-nitrotyrosine
AGEs	Advanced glycation end products
ACC	Acetyl-coa Carboxylase
AGEs	Advanced glycation end products
Akt	Protein kinase B
ALT	Alanine aminotransferase
AMC	Anti-diabetic monomer combination
ARE	Antioxidant response element
Arg-1	Arginase-1
ASC	Apoptosis-associated Speck-like protein containing a caspase recruitment domain
a-SMA	Alpha-Smooth Muscle Actin
AST	Aspartate aminotransferase
Bax	Bcl-2-associated X protein
Bcl-2	B-cell lymphoma 2
BRB	Blood-retinal barrier
CaMK	Calcium/calmodulin-dependent protein kinase
Caspase	Cysteine-dependent Aspartate-specific Protease
CAT	Catalase
CD31	Cluster of Differentiation 31
CD86	Cluster of Differentiation 86
CGRP	Calcitonin gene-related peptide
CHOP	CCAAT/enhancer-binding protein Homologous Protein
Col-I	Collagen type i
Col-VI	Collagen type vi
CREB	Camp Response Element-Binding Protein
CRISPR-Cas9	Clustered Regularly Interspaced Short Palindromic Repeats-CRISPR-associated protein 9
CXCR2	C-x-c motif chemokine receptor 2
CYP2E1	Cytochrome p450 family 2 subfamily e member 1
DFU	Diabetic foot ulcers
DLI	Diabetic liver injury
DN	Diabetic nephropathy
DPN	Diabetic peripheral neuropathy
DR	Diabetic retinopathy
DRG	Dorsal root ganglion
EAF	Ethyl acetate extract
EMT	Epithelial-mesenchymal transition
ER	Endoplasmic reticulum
FBG	Fasting blood glucose
FFA	Free fatty acids
GADA	Glutamic acid decarboxylase antibodies
GDM	Gestational diabetes mellitus
GFAP	Glial fibrillary acidic protein
GIP	Glucose-dependent insulinotropic peptide
GLAST	L-glutamate/L-aspartate transporter
GLP-1	Glucagon-like peptide-1
GPX4	Glutathione peroxidase 4
GRP78	Glucose-regulated protein 78
GRP78/Bip	Glucose-regulated protein 78/binding immunoglobulin protein
GS	Glutamine synthase
GSH-PX	Glutathione peroxidas
GSK3β	Glycogen synthase kinase 3 beta
HDL-C	High-density lipoprotein cholesterol
HFD	High-fat diet
HOMA	Homeostasis model assessment
HUVECs	Human umbilical vein endothelial cells
IAA	Insulin antibodies
IBA-1	Ionized calcium binding adaptor molecule 1
ICA	Islet cell antibodies
ICAM-1	Intercellular adhesion molecule 1
ICV	Intracerebroventricular
IKK	IκB kinase
IL	Interleukin
iNOS	Inducible nitric oxide synthase
IRE1α	Inositol-Requiring Enzyme 1 alpha
IRS-1	Insulin receptor substrate-1
IκB	Inhibitor of κB
JAK	Janus kinase
LD50	Lethal dose
LDL-C	Low-density lipoprotein cholesterol
MAPK	Mitogen-activated protein kinase
MCP-1	Monocyte chemoattractant protein-1 (ccl2)
MDA	Malondialdehyde
MMP-9	Matrix metalloproteinase 9
mTOR	Mechanistic target of rapamycin
MyD88	Myeloid differentiation primary response 88
NADPH	Nicotinamide adenine dinucleotide phosphate
NF-kB	Nuclear factor kappa B
NLRP3	NOD-like receptor family pyrin domain containing 3
Nrf2	Nuclear factor erythroid 2-related factor 2
p-4E-BP1	Phosphorylated eukaryotic translation initiation factor 4e-binding protein 1
PEPCK	Phosphoenolpyruvate carboxykinase
PF	Paeoniflorin
PFK	Phosphofructokinase
PI3K	Phosphatidylinositol 3-kinase
PIM2	Proviral Integration site for Moloney murine leukemia virus 2
PIPK	Phosphatidylinositol phosphate kinase
p-IRF3	Phosphorylated Interferon Regulatory Factor 3
PKCβ1	Protein kinase C beta 1
Pmpca	Peptidase mitochondrial processing alpha
PPAR	Peroxisome proliferator-activated receptor
Prx3	Peroxiredoxin 3
p-SGK1	Phosphorylated Serum/Glucocorticoid Regulated Kinase 1
RAGE	Receptor for advanced glycation end-products
ROS	Reactive oxygen species
RPA	Radix paeoniae alba
SIRT1	Sirtuin 1
Smad	Mothers against decapentaplegic homolog
SOCS	Suppressor of cytokine signaling
SOD	Superoxide dismutase
SREBP1	Sterol regulatory element-binding protein 1
SSYX	Shensong yangxin capsule
STAT	Signal transducer and activator of transcription
STZ	Streptozotocin
Sumo	Small Ubiquitin-like Modifier
SYT	Sanye tablet
T1D	Type 1 diabetes
T2D	Type 2 diabetes
TAOC	Total antioxidant capacity
TC	Total cholesterol
TG	Triglycerides
TGF-β1	Transforming growth factor-β1
TLR2/4	Toll-like receptor 2/4
TNF-α	Tumor necrosis factor-α
TRIF	TIR-domain-containing adapter-inducing interferon-β
TRPV1	Transient receptor potential vanilloid 1
Trx2	Thioredoxin
TrxR2	Thioredoxin reductase 2
TXNIP	Thioredoxin-interacting protein
VEGF2	Vascular endothelial growth factor receptor 2
XBP-1	X-box Binding Protein 1
α2-AR	Alpha-2 adrenergic receptor
γGCS	Gamma-Glutamylcysteine Synthetase

## 2 Pathogenesis

### 2.1 Insulin dysfunction

Diabetes is a chronic metabolic disorder marked by high blood glucose levels resulting from inadequate insulin secretion or resistance to insulin action. Pancreatic dysfunction, particularly the impairment of β-cells responsible for insulin secretion, is a pivotal factor in the development of diabetes (MacDonald and Rorsman, 2023; Krause and De Vito, 2023). The islets of Langerhans, alternatively termed pancreatic islets, are microorgans dispersed throughout the pancreas, playing a vital role in maintaining glucose homeostasis (Bader et al., 2016). Under normal physiological conditions, β-cells within the islets secrete insulin in response to elevated blood glucose levels, facilitating glucose uptake and utilization by peripheral tissues, including muscle and adipose tissue, while suppressing hepatic glucose production (Bennet et al., 2016; Ling et al., 2016). However, in diabetic patients, impaired function and a decreased number of pancreatic β-cells result in inadequate insulin secretion or insulin resistance.

#### 2.1.1 Insufficient insulin secretion

Insulin, secreted by the β-cells in the pancreas, plays a vital role in regulating blood glucose levels. Its main function is to facilitate glucose uptake and utilization by muscle and fat cells, thereby lowering blood glucose levels. Diminished function or quantity of β-cells results in reduced insulin secretion, causing persistent elevation of blood glucose levels (Kalogeropoulou et al., 2008).

In Type 1 diabetes (T1D), inadequate insulin secretion results primarily from autoimmune-mediated damage and death of insulin-secreting cells (Beck et al., 2019). This chronic autoimmune disease occurs when the immune system misidentifies β-cells as foreign and generates antibodies and cytotoxic T cells that attack and destroy these cells. Over time, a gradual decline in the number of β-cells in the pancreas leads to inadequate insulin secretion and ultimately hyperglycemia (Skyler, 2023). Differently, Type 2 diabetes (T2D) primarily results from chronic insulin resistance (Santoro and Kahn, 2023). As the body’s insulin demand rises, the pancreas augments insulin secretion to meet the increased need. However, insulin resistance reduces cellular responsiveness to insulin, necessitating increased insulin levels to maintain normal blood glucose levels. Over time, a progressive decline in pancreatic β-cell function may result in inadequate insulin secretion.

#### 2.1.2 Insulin resistance

Insulin resistance is a primary pathogenic mechanism in T2D and a significant factor contributing to inadequate insulin secretion. It denotes a diminished responsiveness of cells to insulin, leading to impaired insulin biological activity (Gerich, 2003). To maintain normal blood glucose levels, increased insulin secretion is required. Primary causes of insulin resistance encompass obesity, unhealthy dietary habits, and physical inactivity. Obesity, particularly abdominal obesity, constitutes a predominant risk factor for insulin resistance. Fat cells serve not only as energy reservoirs but also as sources of various hormones, including tumor necrosis factor-α (TNF-α) and leptin. These hormones can suppress insulin signaling, contributing to insulin resistance (Kirwan et al., 2002).

With worsening insulin resistance, the pancreas must increase insulin secretion to sustain normal blood glucose levels. Prolonged overwork can result in a progressive decline in the function of pancreatic β-cells, ultimately causing inadequate insulin secretion. Additionally, insulin resistance can result in aberrant hepatic glycogen metabolism (Lei et al., 2019). Insulin’s primary function is to inhibit hepatic glycogenolysis and gluconeogenesis. In the presence of insulin resistance, this inhibitory effect diminishes, resulting in heightened hepatic glycogenolysis and elevated glucose release into the bloodstream.

### 2.2 Hepatic glycogen metabolic disorders

The liver is essential for maintaining blood glucose homeostasis through the storage and release of glycogen to regulate blood sugar levels (Zhang et al., 2022a). Insulin’s primary function is to inhibit hepatic glycogenolysis and gluconeogenesis. However, in cases of insulin resistance or inadequate insulin secretion, this inhibitory effect diminishes, resulting in increased glycogenolysis in the liver and elevated glucose release into the bloodstream, leading to higher blood sugar levels.

Insulin inhibits the activity of hepatic glycogen enzymes, including glycogen phosphorylase and glycogen synthase, reducing glycogen breakdown and lowering glucose release into the bloodstream. Moreover, insulin facilitates the conversion of liver glucose into glycogen for storage, reducing blood glucose levels (Villagarcía et al., 2018). Furthermore, insulin suppresses the activity of pivotal enzymes in hepatic gluconeogenesis, including phosphofructokinase and phosphoenolpyruvate carboxykinase (PEPCK), decreasing glucose production from non-carbohydrate sources (Zheng et al., 2016). However, insulin resistance and insufficient insulin secretion lead to reduced insulin inhibition of glycogen enzymes, decreased insulin promotion of glycogen synthesis, increased glucose production from non-carbohydrate substrates, enhanced glycogen breakdown, diminished glycogen storage in the liver, and elevated glucose release into the bloodstream, exacerbating elevated blood sugar levels.

Abnormal hepatic glycogen metabolism significantly contributes to the onset of diabetes and inadequate blood glucose control (Reed et al., 2021). Hyperglycemia exacerbates the damage to pancreatic β-cells, intensifying insulin deficiency and insulin resistance, establishing a vicious cycle. Furthermore, chronic hyperglycemia can result in multiple microvascular complications, including diabetic nephropathy and diabetic retinopathy.

### 2.3 Abnormal intestinal hormones

Intestinal hormones, including glucagon, insulin, glucagon-like peptide-1 (GLP-1), and glucose-dependent insulinotropic polypeptide (GIP), play a critical role in regulating blood glucose and energy metabolism. These hormones work in concert to influence key physiological processes such as appetite control, gastrointestinal motility, insulin secretion, and insulin resistance, ensuring glucose homeostasis (Sandoval and D'Alessio, 2015; Bany et al., 2023). Among them, GLP-1, secreted by intestinal L cells, is particularly important for stimulating insulin release, suppressing glucagon secretion, slowing gastric emptying, and reducing appetite, all of which contribute to lowering blood glucose levels. Additionally, GLP-1 supports pancreatic β-cell function by promoting cell proliferation, differentiation, and survival, thereby preserving insulin production capacity (Caruso et al., 2023). However, in type 2 diabetes, both GLP-1 secretion and activity are impaired, leading to insufficient insulin release and excessive glucagon levels, which exacerbate glucose dysregulation (Bossart et al., 2022).

GIP, another key intestinal hormone, also stimulates insulin secretion and enhances β-cell function. Unlike GLP-1, GIP levels in diabetic patients may remain normal or even increase; however, its ability to enhance insulin secretion is significantly diminished due to reduced β-cell responsiveness. As a result, GIP fails to effectively regulate blood glucose, further contributing to metabolic dysfunction. Beyond their role in glucose metabolism, intestinal hormones also influence appetite and gastrointestinal function. For instance, reduced GLP-1 levels accelerate gastric emptying, causing more rapid postprandial glucose spikes and worsening insulin resistance (Hædersdal et al., 2022).

Intestinal hormone imbalances extend beyond glucose regulation, playing a crucial role in lipid metabolism and inflammation. GLP-1 and GIP not only modulate blood glucose but also influence body weight and energy balance by regulating lipid metabolism pathways (Pathak et al., 2018). GLP-1 enhances fat oxidation, decreases hepatic fat synthesis, and improves lipid homeostasis. However, in individuals with obesity and diabetes, impaired GLP-1 secretion or function contributes to increased appetite, fat accumulation, and disrupted energy metabolism, further exacerbating obesity and insulin resistance (Guardado Mendoza et al., 2015; Derbenev and Zsombok, 2016). Chronic hyperglycemia, insulin resistance, and lipid abnormalities can also induce a persistent inflammatory state, triggering excessive release of pro-inflammatory cytokines such as TNF-α and IL-6. These inflammatory mediators not only impair β-cell function but also suppress GLP-1 secretion from intestinal L cells, further disrupting hormonal balance (Wong et al., 2022). Additionally, inflammation interferes with intestinal hormone receptor signaling, diminishing the effects of GLP-1 and GIP on β-cell function and further impairing glucose homeostasis (Li et al., 2021a).

Addressing intestinal hormone imbalances is essential for improving metabolic health in diabetes. Therapeutic strategies targeting GLP-1 and GIP pathways, such as GLP-1 receptor agonists and dipeptidyl peptidase-4 (DPP-4) inhibitors, have shown promise in restoring glucose regulation and improving metabolic outcomes (Natale et al., 2025). Understanding the interplay between intestinal hormones, lipid metabolism, and inflammation may provide new avenues for the management of diabetes and its associated complications.

### 2.4 Obesity and abnormal lipid metabolism

Obesity is intrinsically connected to abnormal lipid metabolism, with both factors exerting mutual influence and contributing jointly to the pathogenesis of diabetes (Hu et al., 2022). Obesity induces the excessive expansion and dysfunction of adipose tissue, disrupting the normal regulation of lipid metabolism. Adipose tissue serves not only as an energy storage site but also as an endocrine organ capable of secreting various bioactive substances, including leptin, TNF-α, and interleukin-6 (IL-6) (Oikonomou and Antoniades, 2019). Specifically, leptin functions as a hormone that promotes satiety and energy expenditure; reduced leptin secretion can result in increased appetite and excessive energy intake. On the other hand, TNF-α and IL-6 act as pro-inflammatory factors that increase insulin resistance. Their excessive secretion can impair the body’s insulin signaling pathway, thereby exacerbating insulin resistance and elevating blood glucose levels.

Secondly, obesity leads to abnormal lipid metabolism, manifesting as dyslipidemia and increased lipid accumulation in adipose tissue. Individuals with obesity frequently exhibit dyslipidemia, characterized by elevated levels of low-density lipoprotein cholesterol (LDL-C), reduced high-density lipoprotein cholesterol (HDL-C), and increased triglycerides (TG). These lipid abnormalities are risk factors for cardiovascular disease and can further aggravate insulin resistance and the progression of diabetes (Fu et al., 2011). Within adipose tissue, the overaccumulation of lipids triggers inflammatory responses in adipocytes and interferes with the insulin signaling pathway, exacerbating insulin resistance (Song et al., 2023).

### 2.5 Chronic inflammation and oxidative stress

Chronic inflammation and oxidative stress are critical contributors to impaired islet function and insulin sensitivity in diabetes. Low-grade, persistent inflammation is a hallmark of type 2 diabetes, particularly in obese individuals, where infiltrating macrophages in adipose tissue secrete pro-inflammatory mediators. These factors activate signaling pathways such as c-Jun N-terminal kinase (JNK) and IκB kinase, disrupting insulin receptor substrate (IRS) function and impairing insulin signaling (Tang et al., 2021). Additionally, inflammation directly damages pancreatic β-cells, triggering apoptosis and pyroptosis, ultimately leading to reduced insulin secretion. Recent findings highlight the pivotal role of the NLRP3 inflammasome in diabetes progression. Hyperglycemia and dyslipidemia stimulate NLRP3 activation *via* reactive oxygen species (ROS), promoting inflammatory responses and β-cell pyroptosis, thereby worsening glucose metabolism dysregulation (Radmehr et al., 2025).

Oxidative stress further exacerbates diabetes pathogenesis by generating excess ROS through glucose autooxidation and the formation of advanced glycation end products (AGEs) (Khalid et al., 2022). Elevated ROS levels not only damage β-cells but also activate stress kinases that interfere with insulin signaling, aggravating insulin resistance. Notably, inflammation and oxidative stress reinforce each other, creating a self-perpetuating cycle where inflammation induces ROS production, and oxidative stress amplifies inflammatory signaling. Emerging research has explored therapeutic strategies targeting these mechanisms, including peroxisome proliferator-activated receptor (PPAR) modulators to mitigate insulin resistance and inflammation (Qiu et al., 2023). Additionally, interventions such as NLRP3 inflammasome inhibitors, antioxidants, and gut microbiota modulation are gaining attention for their potential in diabetes prevention and treatment. Understanding the intricate interplay between inflammation and oxidative stress may pave the way for novel therapeutic approaches to improve diabetes management.

### 2.6 Self-immune response and genetic factors

The self-immune response is the body’s immune response to its own tissues, including the autoimmune attack on pancreatic β-cells, which is the main pathogenic mechanism of T1D (Nakayasu et al., 2023). Genetic factors largely determine an individual’s susceptibility to diabetes, especially T2D (Velloso et al., 2013).

T1D is an autoimmune-mediated disease characterized by the autoimmune destruction of pancreatic β-cells, resulting in severe insulin deficiency. Studies have shown that various autoantibodies, such as islet cell antibodies (ICA), glutamic acid decarboxylase antibodies (GADA), insulin antibodies (IAA), and unique self-immune responses like T-cell-mediated immune responses, are associated with the onset of T1D (Ilonen et al., 2019). These self-immune responses lead to a gradual reduction of pancreatic β-cells and decreased insulin secretion, thereby causing hyperglycemic symptoms. However, the genetic susceptibility of T2D is particularly evident. Numerous familial and twin studies have shown that the risk of developing T2D is closely related to genetic factors. Multiple gene variants associated with an increased risk of T2D have been discovered, including TCF7L2, PPARG, KCNJ11, etc (Sanghera et al., 2010; Bonnefond et al., 2020; Mansour Aly et al., 2021). These gene variants affect key physiological processes such as insulin secretion, insulin signaling pathways, pancreatic β-cell function, and energy metabolism, thereby increasing the risk of developing T2D.

Genetic factors not only influence the risk of diabetes onset but also affect the clinical manifestations, complications risk, and treatment response of diabetes. Different gene mutations can lead to varied pancreatic β-cell functions and insulin sensitivities, thereby affecting the clinical manifestations and treatment needs of diabetes. At the same time, some gene mutations are closely related to the risk of diabetes complications, such as cardiovascular disease, kidney disease, and eye diseases.

### 2.7 Lifestyle and environment

In contemporary society, the enhancement of living standards and the rapid pace of life have led to an increase in unfavorable lifestyle factors contributing to the rising prevalence of diabetes, including obesity, irregular dietary habits, physical inactivity, and psychological stress.

Obesity and poor dietary habits stand as predominant risk factors for T2D. The swift tempo of modern urban life facilitates the easy access and consumption of high-sugar, high-fat, and high-calorie foods. Prolonged irregular eating patterns result in fat accumulation, consequently expediting the development of insulin resistance and the onset of T2D (Ladher et al., 2023). Furthermore, consistent physical exercise not only aids in weight management but also improves the body’s insulin sensitivity, thereby promoting stable blood sugar levels and reducing the risk of diabetes.

Psychological stress and shifts in lifestyle habits are equally pivotal factors influencing diabetes onset. The escalating competitive pressures in today’s society can induce chronic emotional strain and mental stress, leading to disruptions in the endocrine system and elevated blood sugar levels, thus hastening the progression of insulin resistance and diabetes (Ghosh et al., 2020). The rapid pace of modern life, coupled with excessive reliance on motor vehicles, sedentary behaviors, and limited physical activity, fosters detrimental lifestyle habits that contribute to diabetes onset.

**FIGURE 2:**
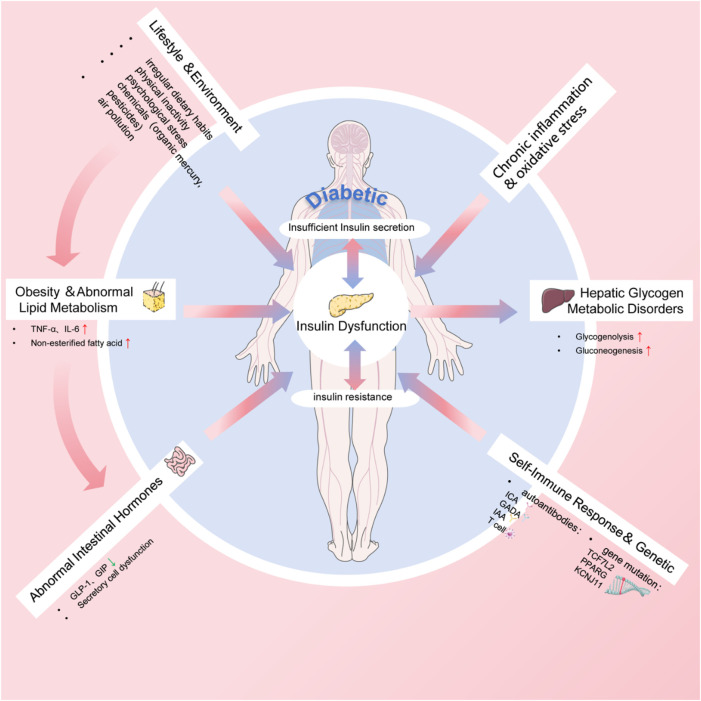
The pathogenesis of diabetes.

Environmental factors are also instrumental in the development and progression of diabetes (Xie et al., 2022). Prolonged exposure to specific chemicals, such as organic mercury and pesticides, has been associated with an elevated risk of diabetes. Moreover, air pollution, particularly extended exposure to fine particulate matter such as PM2.5, correlates with an increased risk of diabetes onset and challenges in blood sugar regulation. These environmental pollutants not only intensify the onset of diabetes but also exacerbate the condition and elevate the risk of complications in individuals with diabetes. In brief, the pathogenesis of diabetes involves multiple factors as shown in Figure 2.

## 3 Pharmacological activities of PF

### 3.1 Diabetes

Diabetes, a chronic metabolic disease caused by insufficient insulin secretion or insulin resistance, characterized primarily by high blood sugar levels (Cole and Florez, 2020). Diabetes is divided into T1D and T2D. T1D is an autoimmune disease resulting from impaired insulin secretion, while T2D is mainly due to insulin resistance and dysfunction of pancreatic β-cells (Hu J. et al., 2023).

Tables 2, 3 summarized the pharmacological effects of PF, along with its extracts, in diabetes mellitus and related complications. PF has gained widespread attention in recent years due to its significant hypoglycemic effects. Studies have shown that PF can enhance glucose utilization, significantly reduce blood glucose levels, and achieve maximum effects 25 min after treatment (Hsu et al., 1997). Another study found that the alcohol extract of Radix Paeoniae Alba (RPA) can significantly reduce fasting blood glucose levels in db/db mice and bring them to levels similar to C57J/B6 mice after 30 days. Furthermore, it effectively improves glucose intolerance, Homeostasis Model Assessment (HOMA) index, *in vivo* glucose uptake, reduces body weight, and alleviates hepatic steatosis (Chang et al., 2016). Importantly, the hypoglycemic effect of PF is independent of insulin action.

**TABLE 2:** Mechanism of PF in diabetes and complications.

Disease	Model Methods	Animal/Cell lines	Dose/ Concentration	Efficacy	Mechanisms	Ref
Diabetes	—	NOD mice	100 mg/kg(i.g.)	Regulate gut microbiota	Anti-Inflammatory, Immunoregulation, ZO-1↑, ZO-2↑, OCCLUDIN↑, IL-6↓, IL-1β↓,Th1/Th17↓, MyD88/TRIF↓	Luo W. et al. (2023)
	STZ 60 mg/kg	Wistar rats	1 mg/kg(i.v.)	Reduce blood sugar	—	Hsu et al. (1997)
	HFD, 12 weeks	C57BL/6J	6.19 mg/kg(i.g.)	Regulate bacteria-cometabolism-inflammation responses	Anti-Inflammatory, Suppress insulin resistance, InsR↑, IRS-2↑, p-Akt↑, TNF-α↓, IL-1β↓, IL-6↓	Han et al. (2020)
DN	STZ 50 mg/kg, 5 days	C57BL/6J	100 mg/kg(i.g.)	Alleviate macrophage infiltration	Anti-Inflammatory, iNOS↓, TNF-α↓, IL-1β↓, MCP-1↓, JAK2/STAT↓	Zhu et al. (2017)
	AGEs 200 μg/mL, 24h	HBZY-1	50 μM	Inhibit Mesangial cell autophagy	Reduce the number of autophagosomes, LC3II/LC3I↓, RAGE↓, Col-IV↓, p-mTOR↑	Chen et al. (2017)
	STZ 65 mg/kg(iv)	SD rats	10 mg/kg(p.o.)	—	Anti-Inflammatory, TGF-β↓, Col-IV↓, ICAM-1↓	Fu et al. (2009)
	STZ 50 mg/kg, 5 days	C57BL/6J	100 mg/kg(i.g.)	Alleviate macrophage infiltration	Anti-Inflammatory, TNF-α↓, IL-1β↓, MCP-1↓, iNOS↓, CD68↓, TLR4↓, NF-κB p65↓, MyD88↓, p-IRAK1↓, Trif↓, p-IRF3↓	Shao et al. (2019)
	STZ 50 mg/kg, 5 days	C57BL/6J, TLR^2−/−^ mice	100 mg/kg(i.p.)	Alleviate macrophage infiltration	Anti-Inflammatory, NF-κB p65↓, TNF-α↓, IL-1β↓, MCP-1↓, MyD88↓, p-IRAK1↓	Shao et al. (2017)
	—	db/m, db/db mice	60 mg/kg(i.p.)	Inhibit macrophage activation	Anti-Inflammatory, iNOS↓, TNF-α↓, IL-1β↓, MCP-1↓, TLR2↓, TLR4↓, MyD88↓, p-IRAK1↓, Trif↓, p-IRF3↓, NF-κB p65↓	Zhang et al. (2017)
DN	Glucose 40 mM, 48 h; STZ 50 mg/kg, 5 days	MPC5; C57BL/6J	40 μM; 200 mg/kg(i.p.)	Inhibit apoptosis	Anti-Inflammatory, TNF-α↓, IL-1β↓, WT-1↑, SYNPO↑, p-MLKL↓, RIPK1/RIPK3↓	Wang et al. (2022b)
	Glucose 1 g/L	HBZY-1	10^−6^ - 10^−3^ M	—	Anti-oxidative stress, Anti-Inflammatory, IL-6↓, MCP-1↓	Zhang et al. (2013)
	Glucose 30 mM, 48 h	Mouse mesangial cells	200 μM	—	Anti-oxidative stress, NADPH↓, ROS↓. TGF-β↓, fibronectin↓	Sun et al. (2015)
	Glucose 30 mM, 48 h	BMDMs	10^-8^ - 10^-4^ M	—	Anti-Inflammatory, TLR2↓, iNOS↓, MyD88↓, p-IRAK1↓, Trif↓, NF-κB p65↓, p-IRF3↓, IRF3↓	Shao et al. (2016)
	Glucose 40 mM, 48 h; STZ 50 mg/kg, 5 days	MPC5; C57BL/6J	80 μM; 200 mg/kg(i.p.)	Restore autophagy	VEGF2↓, caspase 3↓, PI3K/AKT↓	Wang et al. (2022c)
DPN	STZ 150 mg/kg	ddY mice	30 mg/kg(p.o.)	Modulate spinal nociceptive transmission	Activate α2-adrenoceptors, Noradrenaline↑	Lee et al. (2011)
	Glucose 150 mM, 48 h	RSC96	100 μM	Inhibit apoptosis	Anti-oxidative stress, Nrf2/ARE↑, ROS↓, MDA↓, GST↑, GPX↑, Bcl-2↑, Bax↓, Caspase-3↓	Yang et al. (2015)
	Glucose 150 mM, 24 h	RSC96	10 μM	Restoration of mitochondrial function	Pmpca↑, Sumo1↑, Trx2↑, TrxR2↑, Prx3↑, ROS↓	Yang J. et al. (2022)
DPN	Glucose 150 mM, 24 h	SC line	100 μM	Inhibit ER stress and DRGn apoptosis	JNK↓, CHOP↓, XBP1s↑, IRE1α↓, GRP78↓, Bcl-2↑, Bax↓, Caspase-12↓, Caspase-3↓	Zhu et al. (2021)
Diabetic wounds	HG MEM, 48 h; STZ 35 mg/kg	HaCaT; SD rats	100 μM; 30 mg/kg(i.g.)	Promote proliferation and migration, Inhibit apoptosis	Anti-oxidative stress, Nrf2 pathway↑, VEGF↑, TGF-β1↑	Sun et al. (2020)
	HG MEM, 48 h; STZ 35 mg/kg	HaCaT; SD rats	100 μM; 30 mg/kg(i.g.)	—	Anti-Inflammatory, IL-1β↓, IL-18↓, TNF-α↓, CXCR2↓, NF-κB↓, lκB↑, NLRP3↓, Caspase-1↓	Sun et al. (2021)
	STZ 55 mg/kg, 5 days	C57BL6/J	500 μM, hydrogel	Promote proliferation and maturation;	Anti-Inflammatory, CD86↓, TNF-α↓, IL-1β↓, iNOS↓, CD206↑, IL-10↑, TGF-β↑, Arg-1↑, CD31↑, VEGF↑, α-SMA↑, Col-I↑	Yang et al. (2021)
	STZ 60 mg/kg	SD rats	20 mg/mL, hydrogel	Promote angiogenesis, collagen deposition;	Anti-Inflammatory, IL-10↑, TNF-α↓, CD31↑	Guo et al. (2022)
NAFLD	HFD, 12 weeks	C57BL/6J	100 mg/kg(i.g.)	—	Anti-Inflammatory, Triglyceride↓, Cholesterol↓, AST↓, TNF-α↓	Zhou et al. (2019)
	HFD, 10 weeks	SD rats	20 mg/kg(i.g.)	Regulate lipid metabolism	Anti-oxidative stress, TG↓, TC↓, FFA↓, FBG, ALT↓, AST↓, SOD↓, MDA↓, CYP2E1↓, ROS↓, IRS/Akt/GSK3β	Ma et al. (2017a)
DLI	Glucose 300 mM, 24 h; —	AML12; db/db, db/m mice	16 μM; 100 mg/kg(i.g.)	Inhibit apoptosis	Anti-Inflammatory, Anti-oxidative stress, IL-1β↓, IL-18↓, TNF-α↓, F4/80↓, ROS↓, ASC↓, Caspase-1↓, TXNIP/NLRP↓	Wang et al. (2022d)
Diabetic myocardial ischemic injury	STZ 150 mg/kg	C57BL/6, TRPV1^-/-^mice	140 mg/kg(i.g.)	—	TRPV1/CaMK/CREB/CGRP↑	Han et al. (2016)
	STZ 30 mg/kg	SD rats	90 mg/kg(i.g.)	Inhibit apoptosis	Anti-oxidative stress, SOD↑, MDA↑, Bax↓, Bcl-2↑, Caspase-3↓	Li et al. (2021b)
DR	Glucose 25 mM, 6 hours; STZ 60 mg/kg	BV2; CD-1 mice	10 μM; 40 mg/kg(p.o.)	—	Anti-Inflammatory, SOCS3↑, MMP-9↓, IBA-1↓, IL-1β↓, TLR4/p38/NF-κB↓	Pei et al. (2023)
	STZ 50 mg/kg	SD rats	20 mg/kg(i.g.)	Reduce blood sugar, Glial cells activation	GAFP↓, GLAST↑, GS↑	Zhang T. et al. (2019)
	STZ 50 mg/kg	SD rats	4 mg/kg(i.p.)	—	Anti-Inflammatory, GAFP↓, IL-6↓, IL-1↓, TNF-α↓, JAK2/STAT3↓	Zhang et al. (2023a)
Diabetic vascular disease	Glucose 25 mM, 24 h; STZ 30 mg/kg	HUVECs; SD rats	100 μM; 10 mg/kg(i.g.)	Inhibit apoptosis	Anti-Inflammatory, TNF-α↓, PKCβ1↓	Wang et al. (2019)
	STZ 35 mg/kg	C57BL/6J	100 mg/kg(i.g.)	—	Anti-oxidative stress, ALT↓, AST↓, AGEs↓	Huang et al. (2022)
Cognitive dysfunction	STZ 35 mg/kg	SD rats	30 mg/kg(i.g.)	Inhibit tau hyperphosphorylation	Anti-Inflammatory, IL-1β↓, TNF-α↓, SOCS2↓, IRS-1↑, Akt↑, GSK-3β↑	Sun et al. (2017)
	STZ 3 mg/kg, day 1 and 3	C57BL/6	10 mg/kg(i.p.)	Regulate mitochondrial and islet dysfunction	Anti-oxidative stress, p-Pl3K↑, p-Akt↑, p-IRS-1↓	Wang et al. (2018)
Pancreatic β-cells injury	STZ 3 mM, 24 h	INS-1	80 μM	Inhibit apoptosis	Anti-oxidative stress, Caspase-3↓, Bax↓, Bcl-2↑, ROS↓, MDA↓, SOD↑, p-p38↓, p-JNK↓, MAPK↓	Liu C. et al. (2019)
Gestational diabetes	STZ 25 mg/kg	Albino rats	30 mg/kg(p.o.)	Reduce blood sugar	Akt/mTOR↓, p-4E-BP1↓, p-SGK1↓	Zhang et al. (2020)
	STZ 35 mg/kg	SD rats	30 mg/kg(i.g.)	Inhibit ferroptosis	ROS↓, MDA↓, GSH↑, p-PI3K↑, Akt/Nrf2/GPX4↑	Lian et al. (2023)
Diabetic cataract	Glucose 30 mM, 24 h	SRA01/04	10 μM	—	Inhibit EMT of lens epithelial cells, Anti-oxidative stress, E-cadherin↑, N-cadherin↓, Vimentin↓, Snail↓, SOD↑, GSH-Px↑, MDA↓, Bax↓, c-caspase 9↓, cytochrome c↓, Bcl-2↑, SIRT1↑	Zeng et al. (2022)
Diabetic periodontitis	STZ 50 mg/kg	Wistar rats	100 mg/kg(i.g.)	Reduce bone loss, enhance bone quality	Anti-Inflammatory, IL-1β↓, IL-6↓, TNF-α↓	Kuzu et al. (2023)

**TABLE 3:** Mechanism of PF extract and compound in Diabetes and Complications.

Disease	Model methods	Animal/Cell lines	Dose/Dosing method/period	Efficacy	Mechanisms	Ref
Diabetes	HFD 20 weeks	C57BL/6N	SYT 400 mg/kg (i.g.)	Suppress liver lipogenesis	Anti-Inflammatory, TNF-α↓, IL-4↓, IL-6↓	Yao et al. (2021)
	—	db/db mice	PFExt 200 mg/kg (i.g.)	Reduce blood sugar	—	Chang et al. (2016)
	—	db/db mice	EAF 100 mg/kg (i.g.)	Reduce blood sugar	Anti-Inflammatory, Anti-oxidative stress, SOD↑, Catalase↑, MDA↓, ROS↓	Zhang et al. (2023a)
DN	STZ 65 mg/kg	Wistar rats	TGP 200 mg/kg (p.o.)	—	Anti-Inflammatory, Anti-oxidative stress, Col-VI↓, ICAM-1↓, IL-1↓, TNF-α↓, NF-κB p65↓, 3-NT↓, nephrin protein↑, TGF-β1↓	Wu et al. (2009)
	STZ 30 mg/kg	SD rats	MC-TG 808 mg/kg (i.g.)	Ameliorate ER stress-related inflammation	Anti-Inflammatory, GRP78/Bip↓, XBP-1(s)↓, MCP-1↓, ICAM-1↓, p-IRF3↓, NF-κB p-p65↓	Chen et al. (2016)
	STZ 65 mg/kg	Wistar rats	TGP 200 mg/kg (p.o.)	—	Anti-Inflammatory, JAK/STAT3↓	Wang et al. (2012)
	STZ 65 mg/kg	Wistar rats	TGP 200 mg/kg (p.o.)	—	Anti-Inflammatory, α-SMA↓, E-cad↑, TLR2↓	Zhang et al. (2014)
DPN	STZ 60 mg/kg	SD rats	TLN 21.8 g/kg (i.g.)	Improve neurological function	Anti-oxidative stress, TAOC↑, ROS↓, Nrf2↑, γGCS↑, Bcl2↑, Bax↓, Cyto C↓	Ji et al. (2016)
	STZ 60 mg/kg	SD rats	TGP 5.6 mg/kg (i.g.)	Restore mitochondrial function	Pmpca↑, Sumo1↑, Trx2↑, TrxR2↑, Prx3↑, ROS↓	Yang X. et al. (2022)
Diabetic wounds	STZ 35 mg/kg	SD rats	3% PF-SA-gelatin, skin scaffolds	Promote angiogenesis, collagen deposition	Anti-Inflammation, IL-1β↓, CD31↑	Yu et al. (2022)
DLI	—	H4IIE	PR-Et 400 mg/kg	Reduce blood sugar	PEPCK↓	Juan et al. (2010)
Diabetic myocardial fibrosis	STZ 40 mg/kg, 2 days	Wistar rats	SSYX 200 mg/kg	Inhibit cardiac fibrosis	Anti-Fibrosis, TGF-β1↓, Col-I↓, Col-III↓, MMP-2↓, MMP-9↓, α-SMA↓, p-Smad2/3↓, Smad7↑	Shen et al. (2014)
Glycemic variability	Glucose 25 mM, 24 h STZ 30 mg/kg	HUVECs; SD rats	PAE 100 μM PAE 10 mg/kg (i.g.)	Platelet activation	Anti-oxidative stress, CD62p↓, ROS↓, GSH-px↑	Huang et al. (2020)

Research has found that PF can protect NOD mice from the effects of T1D by modulating the gut microbiota and inhibiting the Toll-like receptor 4 (TLR4)-myeloid differentiation primary response protein 88/TIR-domain-containing adapter-inducing interferon-β (MyD88/TRIF) pathway (Zhu et al., 2017). Additionally, a novel anti-diabetic monomer combination (AMC) has been proven to have significant therapeutic effects on high-fat diet (HFD)-induced Type 2 diabetic mice, and its mechanism of action may involve the regulation of bacterial-co-metabolism-inflammatory responses (Han et al., 2020). Sanye Tablet (SYT) is a patented prescription drug rich in PF and widely used for the treatment of T2D and prediabetes. Research has found that SYT can improve lipid metabolism and insulin sensitivity by inhibiting liver lipid synthesis pathways, such as down-regulating Sterol Regulatory Element-Binding Protein 1/Acetyl-CoA Carboxylase (SREBP1/ACC) and Janus Kinase/Transducer and Activator of Transcription (JAK/STAT) signaling pathways, and by modulating the JAK-STAT pathway through its effects on Proviral Integration site for Moloney murine leukemia virus 2 (PIM2) and STAT1 (Chen et al., 2017). Furthermore, the ethyl acetate extract (EAF) of RPA has significant antioxidant and hypoglycemic activities (Zhang et al., 2023c). Overall, PF has a remarkable hypoglycemic effect and may exert its therapeutic effects through various mechanisms, including enhancing insulin secretion, improving insulin resistance, modulating gut microbiota, and antioxidant activities.

### 3.2 DN

**FIGURE 3:**
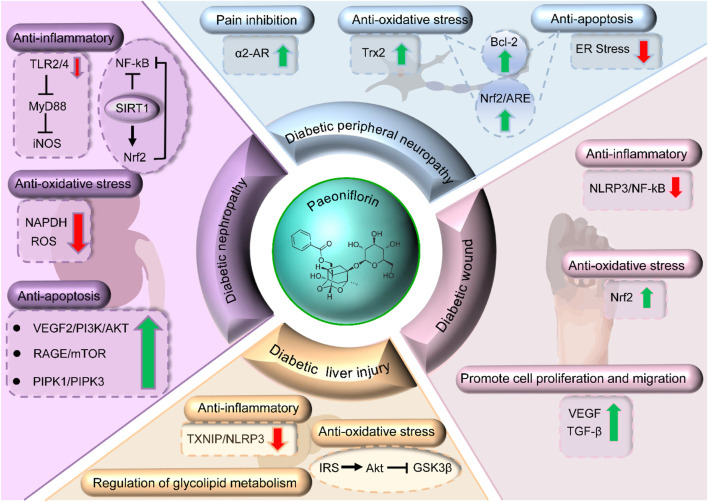
Mechanisms of PF in treating DN, DPR, wound healing, and DLI.

DN is kidney damage caused by diabetes mellitus and is a prevalent complication in diabetic patients (Hu Q. et al., 2023). As diabetes progresses, the glomerular filtration rate decreases, microalbuminuria and proteinuria develop, and may eventually lead to chronic renal failure (Alicic et al., 2017). A large number of studies have confirmed that nephropathy mitigates the effects of PF by inhibiting the inflammatory response, reducing oxidative damage of mesangial cells induced by AGEs, up-regulating autophagy activity and immunomodulation (Figure 3). These cascading events require co-regulation of the multifarious cellular signaling pathways such as JAK2/STAT3 pathway, TLR2/4 pathway, Receptor for Advanced Glycation End products/mammalian target of rapamycin signaling (RAGE/mTOR) pathway, Phosphatidylinositol 3-kinase/Protein Kinase B (PI3K/AKT) pathway, Plant Inositol Phosphate Kinase (PIPK1/PIPK3) pathway, Silent information regulator of transcription 1/Nuclear factor erythroid 2-related factor 2/Nuclear factor kappa B (SIRT1/Nrf2/NF-κB) pathway (Fu et al., 2009; Wu et al., 2009; Wang et al., 2012; Zhang et al., 2014; Chen et al., 2016; Sun et al., 2017; Shao et al., 2017; Zhang et al., 2017; Shao et al., 2019; Wang et al., 2022a).

The study showed that PF and oxypaeniflora were able to attenuate AGEs-induced oxidative damage and inflammation in mesangial cells, thereby improving DN (Zhang et al., 2013). *In vitro* studies, PF reduced the activity of nicotinamide-adenine dinucleotide phosphate oxidase (NADPH), and decreased the level of ROS, transforming growth factor-β1 (TGF-β1) and fibronectin in high glucose cultured mesangial cells (Sun et al., 2015). Although the anti-inflammatory efficacy of PF has been well illustrated in several animal models, whether it could attenuate DN and detailed mechanisms are still obscure. The current study suggested that high glucose activates macrophages primarily through TLR2-dependent mechanisms which aggravated the severity of renal inflammation and eventually contributed to DN. Shao et al. confirmed that PF suppressed HG-induced production of TLR2, activation of downstream signaling and synthesis of inducible nitric oxide synthase (iNOS),and it further inhibited the MyD88-dependent pathway (Shao et al., 2016).

Furthermore, PF has been proven to attenuate AGEs-induced mesangial cell damage, but the regulatory mechanism of autophagy remains unclear. Chen et al. further demonstrated that PF inhibits AGEs-induced mesangial cell dysfunction, at least in part, by inhibiting RAGE and up-regulating p-mTOR levels, thereby inhibiting autophagy and ameliorating DN (Ma et al., 2017a). However, the mechanism of autophagy regulation exerted by PF is also completely different in different cell lines. In MCP5 cells, Wang et al. demonstrated that PF can restore podocyte autophagy and inhibit apoptosis by targeting Vascular Endothelial Growth Factor Receptor 2 (VEGFR2)-mediated the PI3K/AKT pathway to improve DN injury. In addition, VEGFR2 as a target of PF can directly bind to it by molecular docking and surface plasmon resonance detection, which provides a new theoretical basis for PF treatment of DN (Wang et al., 2022b).

### 3.3 Diabetic peripheral neuropathy (DPN)

Among diabetic patients, DPN is prevalent and is characterized by symptoms such as neuralgia, sensory abnormalities, and motor disorders (Hammad et al., 2020; Jensen et al., 2021; Wang et al., 2023b). Recent research suggests that PF, holds promise for DPN treatment. Relevant studies indicate that PF exerts therapeutic effects on DPN through various mechanisms (Lee et al., 2011; Ji et al., 2016). Initially, by activating spinal Alpha-2 adrenergic receptor (α2-AR), PF significantly elevates the pain threshold in diabetic rats, thus alleviating pain sensation (Yang et al., 2015). This discovery provides crucial theoretical underpinnings for employing PF in clinical settings to alleviate DPN-associated pain. Studies have shown that PF can effectively alleviate oxidative stress in Schwann cells induced by high glucose by regulating Nrf2/ARE pathway and Bcl-2-related apoptotic pathway (Yang et al., 2016). Moreover, PF enhances nervous system function in diabetic rats by modulating the expression of mitochondrial thioredoxin (Trx2). Research has shown that PF upregulates Trx2 expression and its associated proteins, diminishes mitochondrial oxidative stress, and consequently mitigates nerve system damage in DPN patients (Yang Y. et al., 2022). Furthermore, PF mitigates nerve cell apoptosis by regulating the endoplasmic reticulum stress pathway, thereby providing additional protection against high glucose-induced nervous system damage (Zhu et al., 2021). Nevertheless, despite evidence of PF’s multifaceted therapeutic effects, its precise mechanism of action remains incompletely understood, necessitating further research to elucidate its molecular mechanisms.

### 3.4 Diabetic wound

Diabetic foot ulcers (DFU) currently pose significant challenges in treatment, with potential severe outcomes including limb ischemia or amputation (Armstrong et al., 2017; Armstrong et al., 2023). PF, possessing biologically active properties such as antioxidants and anti-inflammatory effects, has garnered significant interest (Song et al., 2017; Wen et al., 2019; Zhou et al., 2020; Li et al., 2022; Ren et al., 2023). Research indicates that PF enhances wound healing in diabetic rats by activating the Nrf2 pathway. Consequently, this activation reduces oxidative stress necessary for wound healing, enhances cell proliferation and migration, reduces apoptosis, and upregulates the expression of VEGF and TGF-β1 (Sun et al., 2020). Furthermore, PF can suppress the inflammatory response mediated by NLRP3 and NF-κB, consequently mitigating DFU inflammation and facilitating the healing process (Sun et al., 2021). Recently, there has been a surge in research interest in encapsulating PF in various hydrogels or skin scaffolds for treating DFU. Such encapsulation has demonstrated efficacy in hemostasis, antibacterial activity, modulation of macrophage polarization, inflammation reduction, enhancement of angiogenesis and epithelialization, and promotion of collagen deposition, thereby effectively fostering wound healing (Yang et al., 2021; Guo et l., 2022; Yu et al., 2022). These findings offer crucial experimental validation for the therapeutic potential of PF in managing DFU.

### 3.5 Diabetic liver injury (DLI)

Individuals with diabetes commonly experience DLI, marked by hepatic steatosis, inflammation, and aberrant liver function (Rao et al., 2021; Wang et al., 2023c; Thoudam et al., 2023). DLI can result in severe outcomes, including cirrhosis, liver dysfunction, and potentially liver cancer, significantly reducing patients’ quality of life (Hoofnagle and Björnsson, 2019; Newman et al., 2019; Long et al., 2022). PF exhibits therapeutic potential for diabetic liver disease by inhibiting gluconeogenesis, modulating lipid metabolism, the insulin signaling pathway, and suppressing inflammatory responses (Ma et al., 2017b; Li et al., 2018a; Shu et al., 2019; Zhang et al., 2022b; Wang et al., 2022c; Liu et al., 2023b). Studies indicate that PF reduces blood glucose levels in diabetic rats and db/db mice by inhibiting PEPCK transcription (Juan et al., 2010). Additionally, PF exhibits therapeutic potential for non-alcoholic fatty liver disease (NAFLD) (Zhou et al., 2019). A study suggested that PF inhibits ectopic lipid deposition by modulating lipid metabolism (specifically inhibiting cholesterol synthesis and the *de novo* pathway) and enhancing insulin sensitivity *via* the IRS/Akt/GSK3β insulin signaling pathway and antioxidant mechanisms (Ma et al., 2017c). A recent study validated that PF’s reversal of abnormal liver function and hepatic steatosis in db/db mice is linked to the inhibition of the TXNIP/NLRP3 signaling pathway, indicating TXNIP as a possible target of PF (Wang et al., 2022d). In conclusion, PF exhibits promising therapeutic potential for diabetic liver injury and non-alcoholic fatty liver disease through the modulation of multiple signaling pathways and molecular mechanisms. Further research is warranted to elucidate its therapeutic mechanisms and to conduct clinical trials assessing its efficacy and safety in diabetic patients.

### 3.6 Diabetic myocardial ischemic injury

**FIGURE 4:**
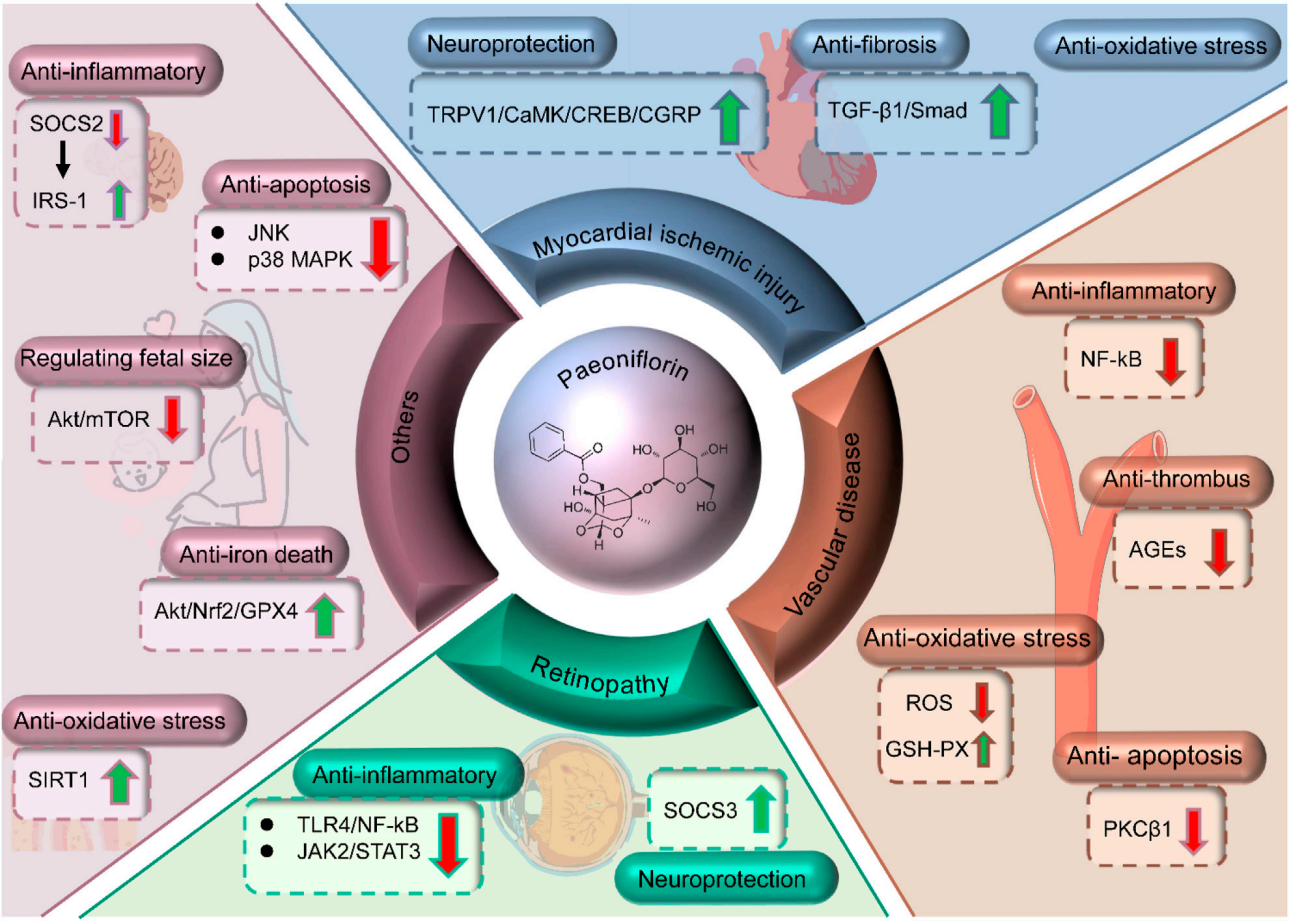
Mechanisms of PF in treating myocardial injury, retinopathy, vascular diseases and others.

Characterized by myocardial fibrosis, inflammatory responses, and impaired cardiac function, diabetic myocardial injury is a prevalent complication in diabetic patients (Figure 4) (Gan et al., 2020; Yu et al., 2021; Wu et al., 2023). Shensong Yangxin Capsule (SSYX), a traditional Chinese herbal medicine abundant in PF, has been extensively used in China for treating arrhythmias. Research indicates that SSYX can mitigate the fibrosis associated with diabetic cardiomyopathy by suppressing the TGF-β1/Mothers against decapentaplegic homolog (Smad) signaling pathway (Shen et al., 2014). Investigations into the therapeutic mechanisms of PF for diabetic myocardial injury predominantly center on its neuroprotective properties and its impact on ischemic heart disease. Notably, PF has been observed to safeguard diabetic mice from myocardial ischemic injury by modulating the transient receptor potential vanilloid 1 (TRPV1)/calcitonin gene-related peptide signaling pathway (Han et al., 2016). Moreover, PF regulates glycolipid metabolism in T2DM model rats, bolsters their antioxidant defenses, suppresses myocardial cell apoptosis, and exhibits a discernible therapeutic effect on myocardial injury (Li et al., 2021b).

### 3.7 Diabetic retinopathy (DR)

DR often leads to vision impairment (Yue et al., 2022; Bryl et al., 2022). Existing research indicates that inflammation of retinal microglial cells is instrumental in the progression of DR (Zhang Y. et al., 2019). Notably, matrix metalloproteinase 9 (MMP-9) in these cells critically compromises the integrity of the blood-retinal barrier and regulates inflammatory processes (Beltramo et al., 2023). Recent studies have demonstrated that PF suppresses the TLR4/NF-κB signaling pathway by enhancing the expression of suppressor of cytokine signaling 3 (SOCS3), leading to a decrease in MMP-9 expression and inflammatory reactions in retinal microglial cells exposed to high glucose (Tu et al., 2019; Fan et al., 2020; Pei et al., 2023). A study suggested that PF could safeguard Müller cells in diabetic rat retinas by upregulating the expression of L-glutamate/L-aspartate transporter (GLAST) and glutamine synthase (GS) (Zhang B. et al., 2019). Subsequently, the research group further highlighted that PF effectively attenuates neuroinflammation in the retinal cells of diabetic rats through the inhibition of the JAK2/STAT3 signaling pathway (Zhang et al., 2023d). Collectively, PF demonstrates pronounced therapeutic potential against diabetic retinopathy by suppressing inflammation, modulating cellular signaling, and reducing blood glucose levels.

### 3.8 Diabetic vascular disease

Diabetes-related vascular complications frequently manifest in diabetic patients. The underlying pathogenesis is predominantly linked to sustained hyperglycemia, endothelial cell impairment due to fluctuations in blood glucose, elevated oxidative stress, and platelet activation (Mohammedi et al., 2015; Yeom et al., 2016; Liu C. et al., 2019; SCORE2-Diabetes Working Group and the ESC Cardiovascular Risk Collaboration, 2023). PF, with its anti-apoptotic, anti-inflammatory, and anti-thrombotic properties, offers substantial protection against cardiovascular and cerebrovascular disorders. Studies have demonstrated that PF can mitigate oxidative stress, diminish inflammatory reactions, and decrease the Protein kinase C beta 1 (PKCβ1) protein expression, thus mitigating damage to human umbilical vein endothelial cells (HUVECs) caused by elevated glucose levels (Wang et al., 2019). In diabetic rat models, PF has proven protective against vascular damage. Furthermore, PF has notable effects in suppressing oxidative stress and platelet activation. It enhances the morphology and viability of HUVECs influenced by glycemic fluctuations, diminishes platelet aggregation, reduces ROS concentrations, and elevates Glutathione peroxidas (GSH-px) levels (Huang et al., 2020). Notably, combined administration of PF and metformin in diabetic rats significantly lowers blood glucose, Alanine Aminotransferase (ALT), and Aspartate Aminotransferase (AST) levels and substantially decreases AGEs, underscoring the therapeutic potential of PF against diabetic vascular complications (Huang et al., 2022). Nevertheless, research investigating the combined therapeutic effects of PF with other conventional antidiabetic medications remains limited, warranting further exploration into their synergistic efficacy and mechanisms.

### 3.9 Others

The therapeutic efficacy of PF in alleviating diabetes-related cognitive dysfunction is well-established. In diabetic rat models, PF ameliorates cognitive impairment by modulating the Suppressor of Cytokine Signaling 2 (SOCS2)/IRS-1 signaling pathway. This enhancement correlates with PF’s capacity to reduce brain inflammatory cytokines, downregulate SOCS2 expression, and boost IRS-1 activity (Sun et al., 2017). Furthermore, PF exhibits neuroprotective properties against cognitive deficits induced by intracerebroventricular (ICV) administration of Streptozotocin (STZ) (Zhang et al., 2020). PF treatment markedly ameliorates STZ-induced mitochondrial dysfunction, augments synaptic density in the hippocampal Cornu Ammonis one region, and improves cerebral insulin signaling by elevating p-PI3K and p-Akt protein levels (Wang et al., 2018). In the context of pancreatic β-cells, PF mitigates STZ-induced apoptosis and safeguards β-cells from STZ-mediated damage by inhibiting the p38 mitogen-activated protein kinase (MAPK) and JNK pathways (Liu Y. et al., 2019). These findings suggest that PF might serve as a natural anti-diabetic agent by alleviating β-cell injury.

Concerning gestational diabetes mellitus (GDM), PF demonstrates potential therapeutic benefits. Research indicates that PF significantly ameliorates blood glucose, leptin, and insulin concentrations and curbs the hyperactivation of the Akt/mTOR signaling in placental tissue. This normalization contributes to fetal size and weight regulation in GDM-affected rats (Zhang et al., 2020). A recent investigation highlighted the protective role of PF in pancreatic tissue of GDM rats, potentially *via* the Akt/Nrf2/GPX4 pathway to counter ferroptosis (Lian et al., 2023). Additionally, PF confers protection against diabetic cataracts. Under hyperglycemic conditions, it substantially enhances the viability of human lens epithelial SRA01/04 cells and suppresses epithelial-mesenchymal transition (EMT) and oxidative damage by upregulating SIRT1 expression (Zeng et al., 2022). In a diabetic periodontitis model, PF demonstrated significant anti-inflammatory effects by reducing pro-inflammatory cytokines, leading to decreased bone loss. Micro-CT analysis further revealed that PF treatment enhanced bone quality by increasing bone mineral density and improving trabecular number and thickness. These results indicate that PF may have potential therapeutic value in managing diabetic periodontitis, particularly through its anti-inflammatory properties and role in preserving bone integrity (Kuzu et al., 2023).

In summary, as a natural monoterpene glycoside, PF presents a multi-faceted therapeutic potential for diabetes and its associated complications. Nevertheless, despite the promising initial findings, further clinical investigations are imperative to validate its safety, efficacy, and elucidate its mechanisms of action.

## 4 Pharmacokinetics of PF

PF, a tetracyclic glycoside predominantly found in plants of the Paeoniaceae family, such as white peony and red peony. Upon entering the human body, peony glycoside quickly reaches the bloodstream *via* routes like oral administration or injection. Following gastrointestinal absorption, it distributes widely in body tissues, including crossing the blood-brain barrier and the placenta (Hu et al., 2016; Wu et al., 2022). In the body, the primary metabolism of peony glycoside occurs in the liver through enzymatic processes, leading to the formation of metabolites that are subsequently excreted by the kidneys (Mei et al., 2023).

After oral administration, PF is rapidly absorbed from the gastrointestinal tract. The absorption process is generally considered to be efficient but may be affected by the presence of other components in the herb or food. It has been reported that PF undergoes passive diffusion across the intestinal membrane (Luo C. et al., 2023). The bioavailability of PF is relatively high, suggesting that it can reach therapeutic concentrations in the bloodstream after oral administration. Once absorbed into the bloodstream, PF is distributed to various tissues and organs. It has a relatively large volume of distribution, indicating that it is extensively distributed in the body. PF is predominantly distributed in the liver, kidneys, and spleen, which are important target organs for its therapeutic effects in diabetes. It is also found in the plasma, where it binds to plasma proteins to some extent (Li et al., 2018b; Li Y. et al., 2023). The distribution of PF to the target organs is essential for its pharmacological action against diabetes. Additionally, PF undergoes extensive metabolism in the liver. The primary metabolic pathway involves hydrolysis of the glucoside bond to form PF aglycone and glucose (Hua et al., 2023). The aglycone can further undergo phase II metabolism, including glucuronidation and sulfation, to form water-soluble metabolites that are excreted in the urine. The metabolism of PF is relatively rapid, with a short half-life, indicating that it may require multiple dosing to maintain therapeutic concentrations in the bloodstream. The primary route of excretion for PF and its metabolites is through the kidneys. Renal excretion accounts for the elimination of the majority of the administered dose within 24 h (Shao et al., 2017). A smaller portion of the dose may also be excreted in the feces. The elimination of PF is relatively rapid, with a clearance rate that is proportional to renal function (Zhai et al., 2016; Chen et al., 2021). Therefore, patients with impaired renal function may require dose adjustment to prevent drug accumulation and potential toxicity.

The pharmacokinetics of PF are characterized by rapid absorption, extensive distribution, rapid metabolism, and renal excretion. Understanding these pharmacokinetic properties is essential for optimizing the therapeutic use of PF in the treatment of diabetes. Further studies are needed to investigate the potential interactions of PF with other drugs and its pharmacokinetics in special populations, such as elderly patients and those with renal impairment.

## 5 Toxicology of PF

Acute toxicity studies have demonstrated that PF exhibits low toxicity (Cai et al., 2021). In rodents, the oral median lethal dose (LD50) is notably high, with rats showing an LD50 of 14.55 g/kg, suggesting a wide safety margin. Even at high oral doses, such as 3 g/kg, no significant adverse reactions or fatalities were observed in animal models (Yu et al., 2022). Additionally, subacute and chronic toxicity studies have been performed to evaluate the long-term effects of PF. Results demonstrate that therapeutic doses (100–400 mg/kg) are generally well tolerated (Zhong et al., 2023). Prolonged use does not lead to significant toxicity or damage to major organs, including the liver and kidneys (Ngo et al., 2019). However, further studies are necessary to explore potential long-term risks, particularly regarding reproductive health, developmental effects, and carcinogenicity.

Genotoxicity and mutagenicity studies were conducted to assess the potential DNA-damaging effects of PF. The results of these studies were generally negative, indicating that PF does not induce significant genetic mutations or chromosomal aberrations (Liu et al., 2006). These findings support the safety of PF in human therapeutic applications. Safety pharmacological studies have confirmed that PF does not produce notable adverse effects on the cardiovascular, respiratory, and central nervous systems (Wang et al., 2016). However, caution should be exercised when administering PF concomitantly with other medications that may affect these systems.

In summary, PF exhibits a favorable toxicological profile with low acute toxicity, minimal subacute and chronic toxicity, and no genotoxic or mutagenic effects. However, continuous monitoring and further studies are necessary to fully elucidate the long-term safety and potential interactions of PF in clinical use.

## 6 Discussion and prospect

Current treatments for diabetes primarily include metformin, sulfonylureas, GLP-1 receptor agonists, DPP-4 inhibitors, and sodium-glucose cotransporter 2 (SGLT2) inhibitors. These drugs regulate blood glucose through various mechanisms. Metformin reduces hepatic glucose output and improves insulin sensitivity (LaMoia and Shulman, 2021), sulfonylureas stimulate insulin secretion from pancreatic β-cells (Lv et al., 2020), GLP-1 receptor agonists and DPP-4 inhibitors enhance incretin activity, and SGLT2 inhibitors lower glucose levels by reducing renal glucose reabsorption (Xie et al., 2023). Despite their efficacy, long-term use of these drugs is associated with limitations such as gastrointestinal discomfort, hypoglycemia, and unresolved safety concerns, necessitating the exploration of alternative therapies.

Natural compounds have gained increasing attention as potential antidiabetic agents due to their multi-target effects and lower incidence of adverse reactions. Among them, plant-derived compounds such as berberine, fenugreek, and baicalin have shown promising hypoglycemic properties. Berberine enhances insulin sensitivity, reduces hepatic glucose production, and modulates gut microbiota by activating the AMP-activated protein kinase (AMPK) pathway (Qin et al., 2020). Fenugreek, rich in saponins and dietary fiber, lowers glucose absorption, enhances insulin secretion, and improves insulin sensitivity (Luo W. et al., 2023). Baicalin exerts antidiabetic effects through its anti-inflammatory, antioxidant, and glucose metabolism-regulating properties (Pan et al., 2021). Compared with these natural compounds, PF demonstrates broader metabolic regulatory potential. In addition to improving insulin resistance, PF protects pancreatic β-cells through anti-inflammatory and antioxidant mechanisms, modulating key pathways such as Nrf2/ARE, NF-κB, and the NLRP3 inflammasome to mitigate oxidative stress and inflammation (Table 1). Furthermore, PF exhibits therapeutic potential in diabetes-related complications, including DN, DNP, and cardiovascular disorders. However, its clinical translation is hindered by poor bioavailability, limiting its therapeutic efficacy.

To overcome this challenge, future research should focus on optimizing PF’s pharmacokinetic properties. Nanotechnology-based strategies, such as nanoemulsions, liposomes, and biopolymer conjugation, could enhance its stability and absorption. Additionally, prodrug development may improve its *in vivo* bioavailability. Given that most current studies are limited to cell cultures and rodent models, more clinically relevant models—such as diabetic large animals and humanized diabetic mice—are needed to better reflect the complexity of human diabetes. Advances in omics technologies, including proteomics, metabolomics, and bioinformatics, can facilitate a deeper understanding of PF’s molecular mechanisms and identify novel therapeutic targets. Furthermore, Clustered Regularly Interspaced Short Palindromic Repeats - CRISPR-associated protein 9 (CRISPR-Cas9) gene editing may provide valuable insights into its precise regulatory pathways.

Despite its promising profile, the long-term safety of PF remains insufficiently characterized. While preclinical studies suggest a favorable safety profile, comprehensive toxicological assessments are necessary before clinical application. Future clinical trials should focus on evaluating its tolerability, optimal dosing, and potential drug interactions in diabetic patients. Additionally, determining the most effective administration route—whether oral, injectable, or transdermal—will be crucial for maximizing therapeutic outcomes.

In conclusion, PF holds significant promise in diabetes management due to its multi-target mechanisms and potential benefits in preventing complications. To facilitate its clinical translation, future studies should integrate advanced drug delivery systems, omics-based analyses, and rigorous clinical trials to optimize its pharmacokinetic profile and ensure safety. These efforts will not only enhance our understanding of PF’s therapeutic potential but may also contribute to the development of novel strategies for diabetes and other metabolic disorders.

## References

[B1] Alicic R. Z. Rooney M. T. Tuttle K. R. (2017). Diabetic kidney disease, challenges, progress, and possibilities. Clin. J. Am. Soc. Nephrol. 12 (12), 2032–2045. 10.2215/CJN.11491116 28522654 PMC5718284

[B2] Armstrong D. G. Boulton A. J. M. Bus S. A. (2017). Diabetic foot ulcers and their recurrence. N. Engl. J. Med. 376 (24), 2367–2375. 10.1056/NEJMra1615439 28614678

[B3] Armstrong D. G. Tan T. W. Boulton A. J. M. Bus S. A. (2023). Diabetic foot ulcers, A review. JAMA 330 (1), 62–75. 10.1001/jama.2023.10578 37395769 PMC10723802

[B4] Bader E. Migliorini A. Gegg M. Moruzzi N. Gerdes J. Roscioni S. S. (2016). Identification of proliferative and mature β-cells in the islets of Langerhans. Nature 535 (7612), 430–434. 10.1038/nature18624 27398620

[B5] Bany Bakar R. Reimann F. Gribble F. M. (2023). The intestine as an endocrine organ and the role of gut hormones in metabolic regulation. Nat. Rev. Gastroenterol. Hepatol. 20 (12), 784–796. 10.1038/s41575-023-00830-y 37626258

[B6] Bayan N. Yazdanpanah N. Rezaei N. (2022). Role of toll-like receptor 4 in diabetic retinopathy. Pharmacol. Res. 175, 105960. 10.1016/j.phrs.2021.105960 34718133

[B7] Beck R. W. Bergenstal R. M. Laffel L. M. Pickup J. C. (2019). Advances in technology for management of type 1 diabetes. Lancet 394 (10205), 1265–1273. 10.1016/S0140-6736(19)31142-0 31533908

[B8] Beltramo E. Mazzeo A. Porta M. (2023). Release of pro-inflammatory/angiogenic factors by retinal microvascular cells is mediated by extracellular vesicles derived from M1-activated microglia. Int. J. Mol. Sci. 25 (1), 15. 10.3390/ijms25010015 38203187 PMC10778795

[B9] Bennet H. Mollet I. G. Balhuizen A. Medina A. Nagorny C. Bagge A. (2016). Serotonin (5-HT) receptor 2b activation augments glucose-stimulated insulin secretion in human and mouse islets of Langerhans. Diabetologia 59 (4), 744–754. 10.1007/s00125-015-3847-6 26733006

[B10] Bonnefond A. Boissel M. Bolze A. Durand E. Toussaint B. Vaillant E. (2020). Pathogenic variants in actionable MODY genes are associated with type 2 diabetes. Nat. Metab. 2 (10), 1126–1134. 10.1038/s42255-020-00294-3 33046911

[B11] Bossart M. Wagner M. Elvert R. Evers A. Hübschle T. Kloeckener T. (2022). Effects on weight loss and glycemic control with SAR441255, a potent unimolecular peptide GLP-1/GIP/GCG receptor triagonist. Cell Metab. 34 (1), 59–74. e10. 10.1016/j.cmet.2021.12.005 34932984

[B12] Bryl A. Mrugacz M. Falkowski M. Zorena K. (2022). The effect of diet and lifestyle on the course of diabetic retinopathy-A review of the literature. Nutrients 14 (6), 1252. 10.3390/nu14061252 35334909 PMC8955064

[B13] Cai T. Wang X. Li B. Xiong F. Wu H. Yang X. (2021). Deciphering the synergistic network regulation of active components from SiNiSan against irritable bowel syndrome via a comprehensive strategy, Combined effects of synephrine, paeoniflorin and naringin. Phytomedicine 86, 153527. 10.1016/j.phymed.2021.153527 33845366

[B14] Caruso I. Marrano N. Biondi G. Genchi V. A. D'Oria R. Sorice G. P. (2023). Glucagon in type 2 diabetes, Friend or foe. Diabetes Metab. Res. Rev. 39 (3), e3609. 10.1002/dmrr.3609 36637256

[B15] Chang C. C. Yuan W. Lin Y. L. Liu R. S. Juan Y. C. Sun W. H. (2016). Evaluation of the *in vivo* therapeutic effects of radix Paeoniae rubra ethanol extract with the hypoglycemic activities measured from multiple cell-based assays. Evid. Based Complement. Altern. Med. 2016, 3262790. 10.1155/2016/3262790 PMC5153506 28018473

[B16] Chen J. Hou X. F. Wang G. Zhong Q. X. Liu Y. Qiu H. H. (2016). Terpene glycoside component from Moutan Cortex ameliorates diabetic nephropathy by regulating endoplasmic reticulum stress-related inflammatory responses. J. Ethnopharmacol. 193, 433–444. 10.1016/j.jep.2016.09.043 27664441

[B17] Chen J. Zhao D. Zhu M. Zhang M. Hou X. Ding W. (2017). Paeoniflorin ameliorates AGEs-induced mesangial cell injury through inhibiting RAGE/mTOR/autophagy pathway. Biomed. Pharmacother. 89, 1362–1369. 10.1016/j.biopha.2017.03.016 28320103

[B18] Chen R. Shen C. Xu Q. Liu Y. Li B. Huang C. (2021). The permeability characteristics and interaction of main components from Si-Ni-San in a MDCK epithelial cell monolayer model. Xenobiotica 51 (2), 239–248. 10.1080/00498254.2017.1359433 28745128

[B19] Cole J. B. Florez J. C. (2020). Genetics of diabetes mellitus and diabetes complications. Nat. Rev. Nephrol. 16 (7), 377–390. 10.1038/s41581-020-0278-5 32398868 PMC9639302

[B20] Derbenev A. V. Zsombok A. (2016). Potential therapeutic value of TRPV1 and TRPA1 in diabetes mellitus and obesity. Semin. Immunopathol. 38 (3), 397–406. 10.1007/s00281-015-0529-x 26403087 PMC4808497

[B21] Fan Q. Guan X. Hou Y. Liu Y. Wei W. Cai X. (2020). Paeoniflorin modulates gut microbial production of indole-3-lactate and epithelial autophagy to alleviate colitis in mice. Phytomedicine 79, 153345. 10.1016/j.phymed.2020.153345 33002829

[B22] Fu J. Li Y. Wang L. Gao B. Zhang N. Ji Q. (2009). Paeoniflorin prevents diabetic nephropathy in rats. Comp. Med. 59 (6), 557–566. 20034431 PMC2798846

[B23] Fu S. Yang L. Li P. Hofmann O. Dicker L. Hide W. (2011). Aberrant lipid metabolism disrupts calcium homeostasis causing liver endoplasmic reticulum stress in obesity. Nature 473 (7348), 528–531. 10.1038/nature09968 21532591 PMC3102791

[B24] Gan L. Xie D. Liu J. Bond Lau W. Christopher T. A. Lopez B. (2020). Small extracellular microvesicles mediated pathological communications between dysfunctional adipocytes and cardiomyocytes as a novel mechanism exacerbating ischemia/reperfusion injury in diabetic mice. Circulation 141 (12), 968–983. 10.1161/CIRCULATIONAHA.119.042640 31918577 PMC7093230

[B25] Gerich J. E. (2003). Contributions of insulin-resistance and insulin-secretory defects to the pathogenesis of type 2 diabetes mellitus. Mayo Clin. Proc. 78 (4), 447–456. 10.4065/78.4.447 12683697

[B26] Ghosh A. Arora B. Gupta R. Anoop S. Misra A. (2020). Effects of nationwide lockdown during COVID-19 epidemic on lifestyle and other medical issues of patients with type 2 diabetes in north India. Diabetes Metab. Syndr. 14 (5), 917–920. 10.1016/j.dsx.2020.05.044 32574982 PMC7265851

[B27] Gorgogietas V. Rajaei B. Heeyoung C. Santacreu B. J. Marín-Cañas S. Salpea P. (2023). GLP-1R agonists demonstrate potential to treat Wolfram syndrome in human preclinical models. Diabetologia 66 (7), 1306–1321. 10.1007/s00125-023-05905-8 36995380 PMC10244297

[B28] Gu J. Chen J. Yang N. Hou X. Wang J. Tan X. (2016). Combination of Ligusticum chuanxiong and Radix Paeoniae ameliorate focal cerebral ischemic in MCAO rats via endoplasmic reticulum stress-dependent apoptotic signaling pathway. J. Ethnopharmacol. 187, 313–324. 10.1016/j.jep.2016.04.024 27108052

[B29] Guardado Mendoza R. Perego C. Finzi G. La Rosa S. Capella C. Jimenez-Ceja L. M. (2015). Delta cell death in the islet of Langerhans and the progression from normal glucose tolerance to type 2 diabetes in non-human primates (baboon, *Papio hamadryas*). Diabetologia 58 (8), 1814–1826. 10.1007/s00125-015-3625-5 26049399 PMC5603258

[B30] Guo C. Wu Y. Li W. Wang Y. Kong Q. (2022). Development of a microenvironment-responsive hydrogel promoting chronically infected diabetic wound healing through sequential hemostatic, antibacterial, and angiogenic activities. ACS Appl. Mater Interfaces 14 (27), 30480–30492. 10.1021/acsami.2c02725 35467827

[B31] Hædersdal S. Lund A. Nielsen-Hannerup E. Maagensen H. Forman J. L. Holst J. J. (2022). The glucagon receptor antagonist LY2409021 does not affect gastrointestinal-mediated glucose disposal or the incretin effect in individuals with and without type 2 diabetes. Eur. J. Endocrinol. 187 (4), 507–518. 10.1530/EJE-22-0291 35977072

[B32] Hammad M. A. Syed Sulaiman S. A. Alghamdi S. Mangi A. A. Aziz N. A. Mohamed Noor D. A. (2020). Statins-related peripheral neuropathy among diabetic patients. Diabetes Metab. Syndr. 14 (4), 341–346. 10.1016/j.dsx.2020.04.005 32305775

[B33] Han F. Zhou D. Yin X. Sun Z. Han J. Ye L. (2016). Paeoniflorin protects diabetic mice against myocardial ischemic injury via the transient receptor potential vanilloid 1/calcitonin gene-related peptide pathway. Cell Biosci. 6, 37. 10.1186/s13578-016-0085-7 27252827 PMC4888521

[B34] Han L. Zhao L. H. Zhang M. L. Li H. T. Gao Z. Z. Zheng X. J. (2020). A novel antidiabetic monomers combination alleviates insulin resistance through bacteria-cometabolism-inflammation responses. Front. Microbiol. 11, 173. 10.3389/fmicb.2020.00173 32132984 PMC7040028

[B35] Hermans M. P. Bouenizabila E. Daniel Amoussou-Guenou K. Jules Gninkoun C. Ahn S. A. Rousseau M. F. (2022). Fatty liver and atherogenic dyslipidemia have opposite effects on diabetic micro- and macrovascular disease. Diabetes Metab. Syndr. 16 (10), 102613. 10.1016/j.dsx.2022.102613 36116326

[B36] Hoofnagle J. H. Björnsson E. S. (2019). Drug-induced liver injury - types and phenotypes. N. Engl. J. Med. 381 (3), 264–273. 10.1056/NEJMra1816149 31314970

[B37] Hsu F. L. Lai C. W. Cheng J. T. (1997). Antihyperglycemic effects of paeoniflorin and 8-debenzoylpaeoniflorin, glucosides from the root of Paeonia lactiflora. Planta Medica 63 (4), 323–325. 10.1055/s-2006-957692 9270377

[B38] Hu J. Ding J. Li X. Li J. Zheng T. Xie L. (2023). Distinct signatures of gut microbiota and metabolites in different types of diabetes, a population-based cross-sectional study. EClinicalMedicine 62, 102132. 10.1016/j.eclinm.2023.102132 37593224 PMC10430172

[B39] Hu P. Y. Liu D. Zheng Q. Wu Q. Tang Y. Yang M. (2016). Elucidation of transport mechanism of paeoniflorin and the influence of ligustilide, senkyunolide I and senkyunolide A on paeoniflorin transport through mdck-mdr1 cells as blood-brain barrier *in vitro* model. Molecules 21 (3), 300. 10.3390/molecules21030300 26950101 PMC6273373

[B40] Hu Q. Jiang L. Yan Q. Zeng J. Ma X. Zhao Y. (2023). A natural products solution to diabetic nephropathy therapy. Pharmacol. Ther. 241, 108314. 10.1016/j.pharmthera.2022.108314 36427568

[B41] Hu W. Yan G. Ding Q. Cai J. Zhang Z. Zhao Z. (2022). Update of Indoles, Promising molecules for ameliorating metabolic diseases. Biomed. Pharmacother. 150, 112957. 10.1016/j.biopha.2022.112957 35462330

[B42] Hua X. Feng X. Hua Y. Wang D. (2023). Paeoniflorin attenuates polystyrene nanoparticle-induced reduction in reproductive capacity and increase in germline apoptosis through suppressing DNA damage checkpoints in *Caenorhabditis elegans* . Sci. Total Environ. 871, 162189. 10.1016/j.scitotenv.2023.162189 36775158

[B43] Huang F. W. Wu W. Q. He Y. Y. Chen J. W. (2022). Effect of paeoniflorin on diabetic macroangiopathy mice. Lingnan J. Emerg. Med. 27 (02), 105–107+111. 10.3969/j.issn.1671-301X.2022.02.002

[B44] Huang Y. Wang J. S. Yang L. Yue L. Zhang L. Zhang Y. H. (2020). Paeoniflorin ameliorates glycemic variability-induced oxidative stress and platelet activation in HUVECs and DM rats. RSC Adv. 10 (69), 42605–42612. 10.1039/d0ra02036b 35692727 PMC9119283

[B45] Ilonen J. Lempainen J. Veijola R. (2019). The heterogeneous pathogenesis of type 1 diabetes mellitus. Nat. Rev. Endocrinol. 15 (11), 635–650. 10.1038/s41574-019-0254-y 31534209

[B46] Jansson Sigfrids F. Dahlström E. H. Forsblom C. Sandholm N. Harjutsalo V. Taskinen M. R. (2021). Remnant cholesterol predicts progression of diabetic nephropathy and retinopathy in type 1 diabetes. J. Intern Med. 290 (3), 632–645. 10.1111/joim.13298 33964025

[B47] Jensen T. S. Karlsson P. Gylfadottir S. S. Andersen S. T. Bennett D. L. Tankisi H. (2021). Painful and non-painful diabetic neuropathy, diagnostic challenges and implications for future management. Brain 144 (6), 1632–1645. 10.1093/brain/awab079 33711103 PMC8320269

[B48] Ji Y. Dou Y. N. Zhao Q. W. Zhang J. Z. Yang Y. Wang T. (2016). Paeoniflorin suppresses TGF-β mediated epithelial-mesenchymal transition in pulmonary fibrosis through a Smad-dependent pathway. Acta Pharmacol. Sin. 37 (6), 794–804. 10.1038/aps.2016.36 27133302 PMC4954768

[B49] Juan Y. C. Tsai W. J. Lin Y. L. Wang G. J. Cheng J. J. Yang H. Y. (2010). The novel anti-hyperglycemic effect of Paeoniae radix via the transcriptional suppression of phosphoenopyruvate carboxykinase (PEPCK). Phytomedicine 17 (8-9), 626–634. 10.1016/j.phymed.2009.12.007 20096551

[B50] Kalogeropoulou D. Lafave L. Schweim K. Gannon M. C. Nuttall F. Q. (2008). Leucine, when ingested with glucose, synergistically stimulates insulin secretion and lowers blood glucose. Metabolism 57 (12), 1747–1752. 10.1016/j.metabol.2008.09.001 19013300

[B51] Khalid M. Petroianu G. Adem A. (2022). Advanced glycation end products and diabetes mellitus: mechanisms and perspectives. Biomolecules 12 (4), 542. 10.3390/biom12040542 35454131 PMC9030615

[B52] Kirwan J. P. Hauguel-De Mouzon S. Lepercq J. Challier J. C. Huston-Presley L. Friedman J. E. (2002). TNF-alpha is a predictor of insulin resistance in human pregnancy. Diabetes 51 (7), 2207–2213. 10.2337/diabetes.51.7.2207 12086951

[B53] Krause M. De Vito G. (2023). Type 1 and type 2 diabetes mellitus, commonalities, differences and the importance of exercise and nutrition. Nutrients 15 (19), 4279. 10.3390/nu15194279 37836562 PMC10574155

[B54] Kuzu T. E. Öztürk K. Gürgan C. A. Yay A. Göktepe Ö. Kantarcı A. (2023). Anti-inflammatory and pro-regenerative effects of a monoterpene glycoside on experimental periodontitis in a rat model of diabetes. J. Periodontal Res. 58 (5), 932–938. 10.1111/jre.13151 37340760

[B55] Ladher N. Hinton R. Veitch E. (2023). Challenges of obesity and type 2 diabetes require more attention to food environment. BMJ. 383 2269. 10.1136/bmj.p2269 37813474 PMC10561015

[B56] LaMoia T. E. Shulman G. I. (2021). Cellular and molecular mechanisms of metformin action. Endocr. Rev. 42 (1), 77–96. 10.1210/endrev/bnaa023 32897388 PMC7846086

[B57] Lee K. K. Omiya Y. Yuzurihara M. Kase Y. Kobayashi H. (2011). Antinociceptive effect of paeoniflorin via spinal α_2_-adrenoceptor activation in diabetic mice. Eur. J. Pain 15 (10), 1035–1039. 10.1016/j.ejpain.2011.04.011 21596599

[B58] Lei Y. Gong L. Tan F. Liu Y. Li S. Shen H. (2019). Vaccarin ameliorates insulin resistance and steatosis by activating the AMPK signaling pathway. Eur. J. Pharmacol. 851, 13–24. 10.1016/j.ejphar.2019.02.029 30779918

[B59] Levy M. Prentice M. Wass J. (2019). Diabetes insipidus. BMJ. 364: l321. 10.1136/bmj.l321 30819684

[B60] Li L. Wang H. Zhao S. Zhao Y. Chen Y. Zhang J. (2022). Paeoniflorin ameliorates lipopolysaccharide-induced acute liver injury by inhibiting oxidative stress and inflammation via SIRT1/FOXO1a/SOD2 signaling in rats. Phytother. Res. 36 (6), 2558–2571. 10.1002/ptr.7471 35570830

[B61] Li R. Andreu-Sánchez S. Kuipers F. Fu J. (2021a). Gut microbiome and bile acids in obesity-related diseases. Best. Pract. Res. Clin. Endocrinol. Metab. 35 (3), 101493. 10.1016/j.beem.2021.101493 33707081

[B62] Li S. S. Tian C. Y. Zhang G. W. Ma L. L. Li J. A. Liu J. X. (2021b). Study on improvement effects and mechanism of paeoniflorin on myocardial injury in type 2 diabetic model rats. China Pharm. 32 (23), 2846–2853. 10.6039/j.issn.1001-0408.2021.23.06

[B63] Li X. Sun C. Zhang J. Hu L. Yu Z. Zhang X. (2023). Protective effects of paeoniflorin on cardiovascular diseases, A pharmacological and mechanistic overview. Front. Pharmacol. 14, 1122969. 10.3389/fphar.2023.1122969 37324475 PMC10267833

[B64] Li Y. Liu Y. Liu S. Gao M. Wang W. Chen K. (2023). Diabetic vascular diseases, molecular mechanisms and therapeutic strategies. Signal Transduct. Target Ther. 8 (1), 152. 10.1038/s41392-023-01400-z 37037849 PMC10086073

[B65] Li Y. C. Qiao J. Y. Wang B. Y. Bai M. Shen J. D. Cheng Y. X. (2018b). Paeoniflorin ameliorates fructose-induced insulin resistance and hepatic steatosis by activating LKB1/AMPK and AKT pathways. Nutrients 10 (8), 1024. 10.3390/nu10081024 30081580 PMC6116094

[B66] Li Z. Liu J. Zhang D. Du X. Han L. Lv C. (2018a). Nuciferine and paeoniflorin can be quality markers of Tangzhiqing tablet, a Chinese traditional patent medicine, based on the qualitative, quantitative and dose-exposure-response analysis. Phytomedicine 44, 155–163. 10.1016/j.phymed.2018.02.006 29519686

[B67] Lian L. B. Chen B. Duan B. (2023). Influence of paeoniflorin on ferroptosis in rats with gestational diabetes mellitus by regulating Akt/Nrf2/GPX4 pathway. Chin. J. Birth Health Hered. 31 (01), 22–26. 10.13404/j.cnki.cjbhh.2023.01.001

[B68] Liang D. Liu L. Qi Y. Nan F. Huang J. Tang S. (2024). Jin-Gui-Shen-Qi Wan alleviates fibrosis in mouse diabetic nephropathy via MHC class II. J. Ethnopharmacol. 324, 117745. 10.1016/j.jep.2024.117745 38228231

[B69] Ling Q. Xu X. Wang B. H. Li L. J. Zheng S. (2016). The origin of new-onset diabetes after liver transplantation, liver, islets, or gut? Transplantation 100 (4), 808–813. 10.1097/TP.0000000000001111 26910326

[B70] Liu C. Ge H. M. Liu B. H. Dong R. Shan K. Chen X. (2019). Targeting pericyte-endothelial cell crosstalk by circular RNA-cPWWP2A inhibition aggravates diabetes-induced microvascular dysfunction. Proc. Natl. Acad. Sci. U. S. A. 116 (15), 7455–7464. 10.1073/pnas.1814874116 30914462 PMC6462073

[B71] Liu J. L. Zhang L. Huang Y. Li X. H. Liu Y. F. Zhang S. M. (2023a). Epsin1-mediated exosomal sorting of Dll4 modulates the tubular-macrophage crosstalk in diabetic nephropathy. Mol. Ther. 31 (5), 1451–1467. 10.1016/j.ymthe.2023.03.027 37016580 PMC10188907

[B72] Liu T. Zhuang Z. Wang D. (2023b). Paeoniflorin mitigates high glucose-induced lifespan reduction by inhibiting insulin signaling in *Caenorhabditis elegans* . Front. Pharmacol. 14, 1202379. 10.3389/fphar.2023.1202379 37405055 PMC10315627

[B73] Liu Y. Han J. Zhou Z. Li D. (2019). Paeoniflorin protects pancreatic β cells from STZ-induced damage through inhibition of the p38 MAPK and JNK signaling pathways. Eur. J. Pharmacol. 853, 18–24. 10.1016/j.ejphar.2019.03.025 30880178

[B74] Liu Z. Q. Jiang Z. H. Liu L. Hu M. (2006). Mechanisms responsible for poor oral bioavailability of paeoniflorin, Role of intestinal disposition and interactions with sinomenine. Pharm. Res. 23 (12), 2768–2780. 10.1007/s11095-006-9100-8 17063398

[B75] Long M. T. Noureddin M. Lim J. K. (2022). AGA clinical practice update, diagnosis and management of nonalcoholic fatty liver disease in lean individuals, expert review. Gastroenterology 163 (3), 764–774.e1. 10.1053/j.gastro.2022.06.023 35842345 PMC9398982

[B76] Luo C. Yang D. Hou C. Tan T. Chao C. (2023). Paeoniflorin protects NOD mice from T1D through regulating gut microbiota and TLR4 mediated MyD88/TRIF pathway. Exp. Cell Res. 422 (1), 113429. 10.1016/j.yexcr.2022.113429 36402426

[B77] Luo W. Deng J. He J. Yin L. You R. Zhang L. (2023). Integration of molecular docking, molecular dynamics and network pharmacology to explore the multi-target pharmacology of fenugreek against diabetes. J. Cell. Mol. Med. 27 (14), 1959–1974. 10.1111/jcmm.17787 37257051 PMC10339091

[B78] Lv W. Wang X. Xu Q. Lu W. (2020). Mechanisms and characteristics of sulfonylureas and glinides. Curr. Top. Med. Chem. 20 (1), 37–56. 10.2174/1568026620666191224141617 31884929

[B79] Ma M. Di H. J. Zhang H. Yao J. H. Dong J. Yan G. J. (2017c). Development of phospholipid vesicle-based permeation assay models capable of evaluating percutaneous penetration enhancing effect. Drug Dev. Ind. Pharm. 43 (12), 2055–2063. 10.1080/03639045.2017.1371730 28845697

[B80] Ma Z. Chu L. Liu H. Wang W. Li J. Yao W. (2017a). Beneficial effects of paeoniflorin on non-alcoholic fatty liver disease induced by high-fat diet in rats. Sci. Rep. 7, 44819. 10.1038/srep44819 28300221 PMC5353673

[B81] Ma Z. Liu H. Wang W. Guan S. Yi J. Chu L. (2017b). Paeoniflorin suppresses lipid accumulation and alleviates insulin resistance by regulating the Rho kinase/IRS-1 pathway in palmitate-induced HepG2Cells. Biomed. Pharmacother. 90, 361–367. 10.1016/j.biopha.2017.03.087 28380411

[B82] MacDonald P. E. Rorsman P. (2023). Metabolic messengers, glucagon. Nat. Metab. 5 (2), 186–192. 10.1038/s42255-022-00725-3 36639733 PMC12494092

[B83] Malik R. A. (2020). Diabetic neuropathy, A focus on small fibres. Diabetes Metab. Res. Rev. 36 (Suppl. 1), e3255. 10.1002/dmrr.3255 31828951

[B84] Mansour Aly D. Dwivedi O. P. Prasad R. B. Käräjämäki A. Hjort R. Thangam M. (2021). Genome-wide association analyses highlight etiological differences underlying newly defined subtypes of diabetes. Nat. Genet. 53 (11), 1534–1542. 10.1038/s41588-021-00948-2 34737425

[B85] McAlister E. Kirkby M. Domínguez-Robles J. Paredes A. J. Anjani Q. K. Moffatt K. (2021). The role of microneedle arrays in drug delivery and patient monitoring to prevent diabetes induced fibrosis. Adv. Drug Deliv. Rev. 175, 113825. 10.1016/j.addr.2021.06.002 34111467

[B86] Mei Y. Tong X. Hu Y. Liu W. Wang J. Lv K. (2023). Comparative pharmacokinetics of six bioactive components of Shen-Wu-Yi-Shen tablets in normal and chronic renal failure rats based on UPLC-TSQ-MS/MS. J. Ethnopharmacol. 317, 116818. 10.1016/j.jep.2023.116818 37348793

[B87] Mohammedi K. Bellili-Muñoz N. Marklund S. L. Driss F. Le Nagard H. Patente T. A. (2015). Plasma extracellular superoxide dismutase concentration, allelic variations in the SOD3 gene and risk of myocardial infarction and all-cause mortality in people with type 1 and type 2 diabetes. Cardiovasc Diabetol. 14, 845. 10.1186/s12933-014-0163-2 25855220 PMC4324771

[B88] Nakayasu E. S. Bramer L. M. Ansong C. Schepmoes A. A. Fillmore T. L. Gritsenko M. A. (2023). Plasma protein biomarkers predict the development of persistent autoantibodies and type 1 diabetes 6 months prior to the onset of autoimmunity. Cell Rep. Med. 4 (7), 101093. 10.1016/j.xcrm.2023.101093 37390828 PMC10394168

[B89] Natale P. Green S. C. Tunnicliffe D. J. Pellegrino G. Toyama T. Strippoli G. F. (2025). Glucagon-like peptide 1 (GLP-1) receptor agonists for people with chronic kidney disease and diabetes. Cochrane database Syst. Rev. 2 (2), CD015849. 10.1002/14651858.CD015849.pub2 39963952 PMC11834151

[B90] Newman C. B. Preiss D. Tobert J. A. Jacobson T. A. Page R. L. 2nd Goldstein L. B. (2019). Statin safety and associated adverse events, A scientific statement from the American heart association. Arterioscler. Thromb. Vasc. Biol. 39 (2), e38–e81. 10.1161/ATV.0000000000000073 30580575

[B91] Ngo T. Kim K. Bian Y. Noh H. Lim K. M. Chung J. H. (2019). Antithrombotic effects of paeoniflorin from paeonia suffruticosa by selective inhibition on shear stress-induced platelet aggregation. Int. J. Mol. Sci. 20 (20), 5040. 10.3390/ijms20205040 31614534 PMC6834133

[B92] Oikonomou E. K. Antoniades C. (2019). The role of adipose tissue in cardiovascular health and disease. Nat. Rev. Cardiol. 16 (2), 83–99. 10.1038/s41569-018-0097-6 30287946

[B93] Pan L. Cho K. S. Yi I. To C. H. Chen D. F. Do C. W. (2021). Baicalein, baicalin, and wogonin: protective effects against ischemia-induced neurodegeneration in the brain and retina. Oxidative Med. Cell. Longev. 2021, 8377362. 10.1155/2021/8377362 PMC8263226 34306315

[B94] Pathak P. Xie C. Nichols R. G. Ferrell J. M. Boehme S. Krausz K. W. (2018). Intestine farnesoid X receptor agonist and the gut microbiota activate G-protein bile acid receptor-1 signaling to improve metabolism. Hepatology 68 (4), 1574–1588. 10.1002/hep.29857 29486523 PMC6111007

[B95] Pei X. Tang S. Jiang H. Zhang W. Xu G. Zuo Z. (2023). Paeoniflorin recued hepatotoxicity under zinc oxide nanoparticles exposure via regulation on gut-liver axis and reversal of pyroptosis. Sci. Total Environ. 904, 166885. 10.1016/j.scitotenv.2023.166885 37678520

[B96] Qin S. Tang H. Li W. Gong Y. Li S. Huang J. (2020). AMPK and its activator berberine in the treatment of neurodegenerative diseases. Curr. Pharm. Des. 26 (39), 5054–5066. 10.2174/1381612826666200523172334 32445451

[B97] Qiu Y. Gan M. Wang X. Liao T. Chen Q. Lei Y. (2023). The global perspective on peroxisome proliferator-activated receptor γ (PPARγ) in ectopic fat deposition: a review. Int. J. Biol. Macromol. 253 (Pt 5), 127042. 10.1016/j.ijbiomac.2023.127042 37742894

[B98] Radmehr E. Yazdanpanah N. Rezaei N. (2025). Non-coding RNAs affecting NLRP3 inflammasome pathway in diabetic cardiomyopathy: a comprehensive review of potential therapeutic options. J. Transl. Med. 23 (1), 249. 10.1186/s12967-025-06269-w 40022088 PMC11871836

[B99] Rao Y. Kuang Z. Li C. Guo S. Xu Y. Zhao D. (2021). Gut Akkermansia muciniphila ameliorates metabolic dysfunction-associated fatty liver disease by regulating the metabolism of L-aspartate via gut-liver axis. Gut Microbes 13 (1), 1–19. 10.1080/19490976.2021.1927633 PMC8158032 34030573

[B100] Reed D. Kumar D. Kumar S. Raina K. Punia R. Kant R. (2021). Transcriptome and metabolome changes induced by bitter melon (Momordica charantia)- intake in a high-fat diet induced obesity model. J. Tradit. Complement. Med. 12 (3), 287–301. 10.1016/j.jtcme.2021.08.011 35493312 PMC9039170

[B101] Ren S. Wang Y. Zhang Y. Yan P. Xiao D. Zhao Y. (2023). Paeoniflorin alleviates AngII-induced cardiac hypertrophy in H9c2 cells by regulating oxidative stress and Nrf2 signaling pathway. Biomed. Pharmacother. 165, 115253. 10.1016/j.biopha.2023.115253 37542855

[B102] Sandoval D. A. D'Alessio D. A. (2015). Physiology of proglucagon peptides, role of glucagon and GLP-1 in health and disease. Physiol. Rev. 95 (2), 513–548. 10.1152/physrev.00013.2014 25834231

[B103] Sanghera D. K. Demirci F. Y. Been L. Ortega L. Ralhan S. Wander G. S. (2010). PPARG and ADIPOQ gene polymorphisms increase type 2 diabetes mellitus risk in Asian Indian Sikhs, Pro12Ala still remains as the strongest predictor. Metabolism 59 (4), 492–501. 10.1016/j.metabol.2009.07.043 19846176 PMC2843807

[B104] Santoro A. Kahn B. B. (2023). Adipocyte regulation of insulin sensitivity and the risk of type 2 diabetes. N. Engl. J. Med. 388 (22), 2071–2085. 10.1056/NEJMra2216691 37256977

[B105] SCORE2-Diabetes Working Group and the ESC Cardiovascular Risk Collaboration (2023). SCORE2-Diabetes 10-year cardiovascular risk estimation in type 2 diabetes in Europe. Eur. Heart J. 44 (28), 2544–2556. 10.1093/eurheartj/ehad260 37247330 PMC10361012

[B106] Shao Y. X. Gong Q. Qi X. M. Wang K. Wu Y. G. (2019). Paeoniflorin ameliorates macrophage infiltration and activation by inhibiting the TLR4 signaling pathway in diabetic nephropathy. Front. Pharmacol. 10, 566. 10.3389/fphar.2019.00566 31191309 PMC6540689

[B107] Shao Y. X. Xu X. X. Li Y. Y. Qi X. M. Wang K. Wu Y. G. (2016). Paeoniflorin inhibits high glucose-induced macrophage activation through TLR2-dependent signal pathways. J. Ethnopharmacol. 193, 377–386. 10.1016/j.jep.2016.08.035 27566204

[B108] Shao Y. X. Xu X. X. Wang K. Qi X. M. Wu Y. G. (2017). Paeoniflorin attenuates incipient diabetic nephropathy in streptozotocin-induced mice by the suppression of the Toll-like receptor-2 signaling pathway. Drug Des. Devel Ther. 11, 3221–3233. 10.2147/DDDT.S149504 PMC5687495 29184392

[B109] Shen N. Li X. Zhou T. Bilal M. U. Du N. Hu Y. (2014). Shensong Yangxin Capsule prevents diabetic myocardial fibrosis by inhibiting TGF-β1/Smad signaling. J. Ethnopharmacol. 157, 161–170. 10.1016/j.jep.2014.09.035 25267579

[B110] Shu J. L. Zhang X. Z. Han L. Zhang F. Wu Y. J. Tang X. Y. (2019). Paeoniflorin-6'-O-benzene sulfonate alleviates collagen-induced arthritis in mice by downregulating BAFF-TRAF2-NF-κB signaling, comparison with biological agents. Acta Pharmacol. Sin. 40 (6), 801–813. 10.1038/s41401-018-0169-5 30446734 PMC6786314

[B111] Sims E. K. Carr A. L. J. Oram R. A. DiMeglio L. A. Evans-Molina C. (2021). 100 years of insulin, celebrating the past, present and future of diabetes therapy. Nat. Med. 27 (7), 1154–1164. 10.1038/s41591-021-01418-2 34267380 PMC8802620

[B112] Skyler J. S. (2023). Importance of residual insulin secretion in type 1 diabetes. Lancet Diabetes Endocrinol. 11 (7), 443–444. 10.1016/S2213-8587(23)00149-3 37290467

[B113] Song S. Xiao X. Guo D. Mo L. Bu C. Ye W. (2017). Protective effects of Paeoniflorin against AOPP-induced oxidative injury in HUVECs by blocking the ROS-HIF-1α/VEGF pathway. Phytomedicine 34, 115–126. 10.1016/j.phymed.2017.08.010 28899493

[B114] Song W. Postoak J. L. Yang G. Guo X. Pua H. H. Bader J. (2023). Lipid kinase PIK3C3 maintains healthy brown and white adipose tissues to prevent metabolic diseases. Proc. Natl. Acad. Sci. U. S. A. 120 (1), e2214874120. 10.1073/pnas.2214874120 36574710 PMC9910429

[B115] Sun M. Huang L. Zhu J. Bu W. Sun J. Fang Z. (2015). Screening nephroprotective compounds from cortex Moutan by mesangial cell extraction and UPLC. Arch. Pharm. Res. 38 (6), 1044–1053. 10.1007/s12272-014-0469-3 25156360

[B116] Sun X. Li S. Xu L. Wang H. Ma Z. Fu Q. (2017). Paeoniflorin ameliorates cognitive dysfunction via regulating SOCS2/IRS-1 pathway in diabetic rats. Physiol. Behav. 174, 162–169. 10.1016/j.physbeh.2017.03.020 28322909

[B117] Sun X. Wang X. Zhao Z. Chen J. Li C. Zhao G. (2020). Paeoniflorin accelerates foot wound healing in diabetic rats though activating the Nrf2 pathway. Acta histochem. 122 (8), 151649. 10.1016/j.acthis.2020.151649 33166863

[B118] Sun X. Wang X. Zhao Z. Chen J. Li C. Zhao G. (2021). Paeoniflorin inhibited nod-like receptor protein-3 inflammasome and NF-κB-mediated inflammatory reactions in diabetic foot ulcer by inhibiting the chemokine receptor CXCR2. Drug Dev. Res. 82 (3), 404–411. 10.1002/ddr.21763 33236457

[B119] Tang G. Li S. Zhang C. Chen H. Wang N. Feng Y. (2021). Clinical efficacies, underlying mechanisms and molecular targets of Chinese medicines for diabetic nephropathy treatment and management. Acta Pharm. Sin. B 11 (9), 2749–2767. 10.1016/j.apsb.2020.12.020 34589395 PMC8463270

[B120] Thoudam T. Chanda D. Lee J. Y. Jung M. K. Sinam I. S. Kim B. G. (2023). Enhanced Ca2+-channeling complex formation at the ER-mitochondria interface underlies the pathogenesis of alcohol-associated liver disease. Nat. Commun. 14 (1), 1703. 10.1038/s41467-023-37214-4 36973273 PMC10042999

[B121] Tokarz V. L. MacDonald P. E. Klip A. (2018). The cell biology of systemic insulin function. J. Cell Biol. 217 (7), 2273–2289. 10.1083/jcb.201802095 29622564 PMC6028526

[B122] Tu J. Guo Y. Hong W. Fang Y. Han D. Zhang P. (2019). The regulatory effects of paeoniflorin and its derivative paeoniflorin-6'-O-benzene sulfonate CP-25 on inflammation and immune diseases. Front. Pharmacol. 10, 57. 10.3389/fphar.2019.00057 30804784 PMC6370653

[B123] Velloso L. A. Eizirik D. L. Cnop M. (2013). Type 2 diabetes mellitus--an autoimmune disease? Nat. Rev. Endocrinol. 9 (12), 750–755. 10.1038/nrendo.2013.131 23835371

[B124] Villagarcía H. G. Román C. L. Castro M. C. González L. A. Ronco M. T. Francés D. E. (2018). Liver carbohydrates metabolism, A new islet-neogenesis associated protein peptide (INGAP-PP) target. Peptides 101, 44–50. 10.1016/j.peptides.2018.01.001 29305881

[B125] Wang A. Gong Y. Pei Z. Jiang L. Xia L. Wu Y. (2022a). Paeoniflorin ameliorates diabetic liver injury by targeting the TXNIP-mediated NLRP3 inflammasome in db/db mice. Int. Immunopharmacol. 109, 108792. 10.1016/j.intimp.2022.108792 35483236

[B126] Wang C. Yuan J. Zhang L. L. Wei W. (2016). Pharmacokinetic comparisons of Paeoniflorin and Paeoniflorin-6'O-benzene sulfonate in rats via different routes of administration. Xenobiotica 46 (12), 1142–1150. 10.3109/00498254.2016.1149633 26999037

[B127] Wang D. Liu L. Li S. Wang C. (2018). Effects of paeoniflorin on neurobehavior, oxidative stress, brain insulin signaling, and synaptic alterations in intracerebroventricular streptozotocin-induced cognitive impairment in mice. Physiol. Behav. 191, 12–20. 10.1016/j.physbeh.2018.03.016 29572012

[B128] Wang J. S. Huang Y. Zhang S. Yin H. J. Zhang L. Zhang Y. H. (2019). A protective role of paeoniflorin in fluctuant hyperglycemia-induced vascular endothelial injuries through antioxidative and anti-inflammatory effects and reduction of PKCβ1. Oxid. Med. Cell Longev. 2019, 5647219. 10.1155/2019/5647219 31093316 PMC6481012

[B129] Wang K. Wu Y. G. Su J. Zhang J. J. Zhang P. Qi X. M. (2012). Total glucosides of paeony regulates JAK2/STAT3 activation and macrophage proliferation in diabetic rat kidneys. Am. J. Chin. Med. 40 (3), 521–536. 10.1142/S0192415X12500401 22745068

[B130] Wang M. Zhang Q. Lou S. Jin L. Wu G. Wu W. (2023a). Inhibition of MD2 by natural product-drived JM-9 attenuates renal inflammation and diabetic nephropathy in mice. Biomed. Pharmacother. 168, 115660. 10.1016/j.biopha.2023.115660 37806092

[B131] Wang T. Shan M. Y. Tang C. Y. Cheng M. Y. Chen B. Yan J. (2023b). Linarin ameliorates diabetic liver injury by alleviating oxidative stress and inflammation through the inhibition of AKR1B1. Comb. Chem. High. Throughput Screen 26. 10.2174/1386207326666230412084201 37046194

[B132] Wang W. Ji Q. Ran X. Li C. Kuang H. Yu X. (2023c). Prevalence and risk factors of diabetic peripheral neuropathy, A population-based cross-sectional study in China. Diabetes Metab. Res. Rev. 39 (8), e3702. 10.1002/dmrr.3702 37490047

[B133] Wang X. Jiang L. Liu X. Q. Huang Y. B. Wang A. L. Zeng H. X. (2022b). Paeoniflorin binds to VEGFR2 to restore autophagy and inhibit apoptosis for podocyte protection in diabetic kidney disease through PI3K-AKT signaling pathway. Phytomedicine 106, 154400. 10.1016/j.phymed.2022.154400 36049428

[B134] Wang X. Liu X. Q. Jiang L. Huang Y. B. Zeng H. X. Zhu Q. J. (2022c). Paeoniflorin directly binds to TNFR1 to regulate podocyte necroptosis in diabetic kidney disease. Front. Pharmacol. 13, 966645. 10.3389/fphar.2022.966645 36147345 PMC9486100

[B135] Wang Y. You K. You Y. Li Q. Feng G. Ni J. (2022d). Paeoniflorin prevents aberrant proliferation and differentiation of intestinal stem cells by controlling C1q release from macrophages in chronic colitis. Pharmacol. Res. 182, 106309. 10.1016/j.phrs.2022.106309 35716915

[B136] Wen J. Xu B. Sun Y. Lian M. Li Y. Lin Y. (2019). Paeoniflorin protects against intestinal ischemia/reperfusion by activating LKB1/AMPK and promoting autophagy. Pharmacol. Res. 146, 104308. 10.1016/j.phrs.2019.104308 31181335

[B137] Wong C. K. Yusta B. Koehler J. A. Baggio L. L. McLean B. A. Matthews D. (2022). Divergent roles for the gut intraepithelial lymphocyte GLP-1R in control of metabolism, microbiota, and T cell-induced inflammation. Cell Metab. 34 (10), 1514–1531.e7. 10.1016/j.cmet.2022.08.003 36027914

[B138] Wu H. Zhang P. Zhou J. Hu S. Hao J. Zhong Z. (2024). Paeoniflorin confers ferroptosis resistance by regulating the gut microbiota and its metabolites in diabetic cardiomyopathy. Am. J. Physiol. Cell Physiol. 326 (3), C724–C741. 10.1152/ajpcell.00565.2023 38223927

[B139] Wu J. Zhang D. Hu L. Zheng X. Chen C. (2022). Paeoniflorin alleviates NG-nitro-L-arginine methyl ester (L-NAME)-induced gestational hypertension and upregulates silent information regulator 2 related enzyme 1 (SIRT1) to reduce H2O2-induced endothelial cell damage. Bioengineered 13 (2), 2248–2258. 10.1080/21655979.2021.2024325 35030965 PMC8973614

[B140] Wu Y. Huang T. Li X. Shen C. Ren H. Wang H. (2023). Retinol dehydrogenase 10 reduction mediated retinol metabolism disorder promotes diabetic cardiomyopathy in male mice. Nat. Commun. 14 (1), 1181. 10.1038/s41467-023-36837-x 36864033 PMC9981688

[B141] Wu Y. Ren K. Liang C. Yuan L. Qi X. Dong J. (2009). Renoprotective effect of total glucosides of paeony (TGP) and its mechanism in experimental diabetes. J. Pharmacol. Sci. 109 (1), 78–87. 10.1254/jphs.08112fp 19151544

[B142] Xie J. Wang M. Long Z. Ning H. Li J. Cao Y. (2022). Global burden of type 2 diabetes in adolescents and young adults 1990-2019, systematic analysis of the Global Burden of Disease Study 2019. BMJ 379, e072385. 10.1136/bmj-2022-072385 36740855 PMC9727920

[B143] Xie Y. Bowe B. Xian H. Loux T. McGill J. B. Al-Aly Z. (2023). Comparative effectiveness of SGLT2 inhibitors, GLP-1 receptor agonists, DPP-4 inhibitors, and sulfonylureas on risk of major adverse cardiovascular events: emulation of a randomised target trial using electronic health records. lancet. Diabetes and Endocrinol. 11 (9), 644–656. 10.1016/S2213-8587(23)00171-7 37499675

[B144] Yang H. Song L. Sun B. Chu D. Yang L. Li M. (2021). Modulation of macrophages by a paeoniflorin-loaded hyaluronic acid-based hydrogel promotes diabetic wound healing. Mater Today Bio 12, 100139. 10.1016/j.mtbio.2021.100139 PMC8488309 34632363

[B145] Yang J. Wei Y. Zhao T. Li X. Zhao X. Ouyang X. (2022). Magnolol effectively ameliorates diabetic peripheral neuropathy in mice. Phytomedicine 107, 154434. 10.1016/j.phymed.2022.154434 36122436

[B146] Yang X. Li X. Zhu Y. Gao Y. Xu L. (2022). Paeoniflorin upregulates mitochondrial thioredoxin of Schwann cells to improve diabetic peripheral neuropathy indicated by 4D label-free quantitative proteomics. Oxid. Med. Cell Longev. 2022, 4775645. 10.1155/2022/4775645 35340203 PMC8956397

[B147] Yang X. Yao W. Li Q. Liu H. Shi H. Gao Y. (2015). Mechanism of Tang Luo Ning effect on attenuating of oxidative stress in sciatic nerve of STZ-induced diabetic rats. J. Ethnopharmacol. 174, 1–10. 10.1016/j.jep.2015.07.047 26254599

[B148] Yang X. Yao W. Shi H. Liu H. Li Y. Gao Y. (2016). Paeoniflorin protects Schwann cells against high glucose induced oxidative injury by activating Nrf2/ARE pathway and inhibiting apoptosis. J. Ethnopharmacol. 185, 361–369. 10.1016/j.jep.2016.03.031 26979341

[B149] Yao M. Li L. Huang M. Tan Y. Shang Y. Meng X. (2021). Sanye tablet ameliorates insulin resistance and dysregulated lipid metabolism in high-fat diet-induced obese mice. Front. Pharmacol. 12, 713750. 10.3389/fphar.2021.713750 34658856 PMC8511530

[B150] Yeom E. Byeon H. Lee S. J. (2016). Effect of diabetic duration on hemorheological properties and platelet aggregation in streptozotocin-induced diabetic rats. Sci. Rep. 6, 21913. 10.1038/srep21913 26898237 PMC4762006

[B151] Yu H. Gong W. Mei J. Qin L. Piao Z. You D. (2022). The efficacy of a paeoniflorin-sodium alginate-gelatin skin scaffold for the treatment of diabetic wound, an *in vivo* study in a rat model. Biomed. Pharmacother. 151, 113165. 10.1016/j.biopha.2022.113165 35609370

[B152] Yu L. M. Dong X. Xue X. D. Xu S. Zhang X. Xu Y. L. (2021). Melatonin attenuates diabetic cardiomyopathy and reduces myocardial vulnerability to ischemia-reperfusion injury by improving mitochondrial quality control, Role of SIRT6. J. Pineal Res. 70 (1), e12698. 10.1111/jpi.12698 33016468

[B153] Yue T. Shi Y. Luo S. Weng J. Wu Y. Zheng X. (2022). The role of inflammation in immune system of diabetic retinopathy, Molecular mechanisms, pathogenetic role and therapeutic implications. Front. Immunol. 13, 1055087. 10.3389/fimmu.2022.1055087 36582230 PMC9792618

[B154] Zeng K. Xi W. Qiao Y. Huang X. Liu X. (2022). Paeoniflorin inhibits epithelial mesenchymal transformation and oxidative damage of lens epithelial cells in diabetic cataract via sirtuin 1 upregulation. Bioengineered 3 (3), 5903–5914. 10.1080/21655979.2021.2018534 PMC8974002 35184653

[B155] Zhai T. Sun Y. Li H. Zhang J. Huo R. Li H. (2016). Unique immunomodulatory effect of paeoniflorin on type I and II macrophages activities. J. Pharmacol. Sci. 130 (3), 143–150. 10.1016/j.jphs.2015.12.007 26852260

[B156] Zhang B. Li F. J. Liu X. Z. Zuo Z. F. (2023a). Paeoniflorin inhibits retinal cell inflammation in diabetes rats based on JAK2/STAT3 signaling pathway. Chin. J. Ophthalmol. 33 (04), 305–310. 10.13444/j.cnki.zgzyykzz.2023.04.002

[B157] Zhang B. Li F. J. Zuo Z. S. (2019). Protective effects of paeoniflorin on retinal Müller cells of diabetic rats. Chin. J. Ophthalmol. 29 (01), 5–9. 10.19879/j.cnki.1005-5304.202205845

[B158] Zhang L. Deng M. Wang S. Y. Ding Q. Liu J. H. Xie X. (2023b). Mitigation of Paeoniae Radix Alba extracts on H2O2-induced oxidative damage in HepG2 cells and hyperglycemia in zebrafish, and identification of phytochemical constituents. Front. Nutr. 10, 1135759. 10.3389/fnut.2023.1135759 36908919 PMC9995737

[B159] Zhang L. Han L. Ma J. Wu T. Wei Y. Zhao L. (2022a). Exploring the synergistic and complementary effects of berberine and paeoniflorin in the treatment of type 2 diabetes mellitus by network pharmacology. Eur. J. Pharmacol. 919, 174769. 10.1016/j.ejphar.2022.174769 35151646

[B160] Zhang L. Peng C. Y. Wang P. X. Xu L. Liu J. H. Xie X. (2023c). Hypoglycemic and H2O2-induced oxidative injury protective effects and the phytochemical profiles of the ethyl acetate fraction from Radix Paeoniae Alba. Front. Nutr. 10, 1126359. 10.3389/fnut.2023.1126359 36908916 PMC9998525

[B161] Zhang L. Wei W. (2020). Anti-inflammatory and immunoregulatory effects of paeoniflorin and total glucosides of paeony. Pharmacol. Ther. 207, 107452. 10.1016/j.pharmthera.2019.107452 31836457

[B162] Zhang M. H. Feng L. Zhu M. M. Gu J. F. Wu C. Jia X. B. (2013). Antioxidative and anti-inflammatory activities of paeoniflorin and oxypaeoniflora on AGEs-induced mesangial cell damage. Planta Med. 79 (14), 1319–1323. 10.1055/s-0033-1350649 23881455

[B163] Zhang T. Ouyang H. Mei X. Lu B. Yu Z. Chen K. (2019). Erianin alleviates diabetic retinopathy by reducing retinal inflammation initiated by microglial cells via inhibiting hyperglycemia-mediated ERK1/2-NF-κB signaling pathway. FASEB J. 33 (11), 11776–11790. 10.1096/fj.201802614RRR 31365278 PMC6902687

[B164] Zhang T. Zhu Q. Shao Y. Wang K. Wu Y. (2017). Paeoniflorin prevents TLR2/4-mediated inflammation in type 2 diabetic nephropathy. Biosci. Trends 11 (3), 308–318. 10.5582/bst.2017.01104 28626209

[B165] Zhang W. Zhao L. Su S. Q. Xu X. X. Wu Y. G. (2014). Total glucosides of paeony attenuate renal tubulointerstitial injury in STZ-induced diabetic rats, role of Toll-like receptor 2. J. Pharmacol. Sci. 125 (1), 59–67. 10.1254/jphs.13173fp 24739281

[B166] Zhang X. Yin H. Zhang X. Jiang X. Liu Y. Zhang H. (2022b). N6-methyladenosine modification governs liver glycogenesis by stabilizing the glycogen synthase 2 mRNA. Nat. Commun. 13 (1), 7038. 10.1038/s41467-022-34808-2 36396934 PMC9671881

[B167] Zhang Y. Li Y. Li C. Zhao Y. Xu L. Ma S. (2023d). Paeonia × suffruticosa Andrews leaf extract and its main component apigenin 7-O-glucoside ameliorate hyperuricemia by inhibiting xanthine oxidase activity and regulating renal urate transporters. Phytomedicine 118, 154957. 10.1016/j.phymed.2023.154957 37478683

[B168] Zhang Y. Liang Y. Liu H. Huang Y. Li H. Chen B. (2020). Paeoniflorin attenuates gestational diabetes via Akt/mTOR pathway in a rat model. Food Nutr. Res. 64: 10.29219/fnr.v64.4362 PMC7672451 33240030

[B169] Zheng X. Ke Y. Feng A. Yuan P. Zhou J. Yu Y. (2016). The mechanism by which amentoflavone improves insulin resistance in HepG2 cells. Molecules 21 (5), 624. 10.3390/molecules21050624 27187341 PMC6274486

[B170] Zhong B. L. He S. Q. Sun H. T. Chen W. C. Zhang M. J. Zhong X. D. (2023). Paeoniflorin coordinates macrophage polarization and mitigates liver inflammation and fibrogenesis by targeting the NF-[Formula: see text]B/HIF-1α pathway in CCl_4_-induced liver fibrosis. Am. J. Chin. Med. 51 (5), 1249–1267. 10.1142/S0192415X2350057X 37317554

[B171] Zhou X. Fouda S. Zeng X. Y. Li D. Zhang K. Xu J. (2019). Characterization of the therapeutic profile of albiflorin for the metabolic syndrome. Front. Pharmacol. 10, 1151. 10.3389/fphar.2019.01151 31680948 PMC6797612

[B172] Zhou Y. X. Gong X. H. Zhang H. Peng C. (2020). A review on the pharmacokinetics of paeoniflorin and its anti-inflammatory and immunomodulatory effects. Biomed. Pharmacother. 130, 110505. 10.1016/j.biopha.2020.110505 32682112

[B173] Zhu S. H. Liu B. Q. Hao M. J. Fan Y. X. Qian C. Teng P. (2017). Paeoniflorin suppressed high glucose-induced retinal microglia MMP-9 expression and inflammatory response via inhibition of TLR4/NF-κB pathway through upregulation of SOCS3 in diabetic retinopathy. Inflammation 40 (5), 1475–1486. 10.1007/s10753-017-0571-z 28639050

[B174] Zhu Y. Han S. Li X. Gao Y. Zhu J. Yang X. (2021). Paeoniflorin effect of Schwann cell-derived exosomes ameliorates dorsal root ganglion neurons apoptosis through IRE1α pathway. Evid. Based Complement. Altern. Med. 2021, 6079305. 10.1155/2021/6079305 PMC8490051 34616478

